# Coupled Dynamics of Iron and Phosphorus in Sediments of an Oligotrophic Coastal Basin and the Impact of Anaerobic Oxidation of Methane

**DOI:** 10.1371/journal.pone.0062386

**Published:** 2013-04-23

**Authors:** Caroline P. Slomp, Haydon P. Mort, Tom Jilbert, Daniel C. Reed, Bo G. Gustafsson, Mariette Wolthers

**Affiliations:** 1 Department of Earth Sciences – Geochemistry, Faculty of Geosciences, Utrecht University, Utrecht, The Netherlands; 2 Universidade Federal de Pernambuco, Recife, Brazil; 3 Baltic Nest Institute, Stockholm University, Stockholm, Sweden; 4 Department of Chemistry, University College London, London, United Kingdom; KULeuven, Belgium

## Abstract

Studies of phosphorus (P) dynamics in surface sediments of lakes and coastal seas typically emphasize the role of coupled iron (Fe), sulfur (S) and P cycling for sediment P burial and release. Here, we show that anaerobic oxidation of methane (AOM) also may impact sediment P cycling in such systems. Using porewater and sediment profiles for sites in an oligotrophic coastal basin (Bothnian Sea), we provide evidence for the formation of Fe-bound P (possibly vivianite; Fe_3_(PO_4_)_2_^.^8H_2_O) below the zone of AOM with sulfate. Here, dissolved Fe^2+^ released from oxides is no longer scavenged by sulfide and high concentrations of both dissolved Fe^2+^ (>1 mM) and PO_4_ in the porewater allow supersaturation with respect to vivianite to be reached. Besides formation of Fe(II)-P, preservation of Fe-oxide bound P likely also contributes to permanent burial of P in Bothnian Sea sediments. Preliminary budget calculations suggest that the burial of Fe-bound P allows these sediments to act as a major sink for P from the adjacent eutrophic Baltic Proper.

## Introduction

Phosphorus (P) is an important nutrient in coastal systems. A high availability of P has been shown to contribute to water quality problems such as eutrophication, bottom water anoxia and the occurrence of harmful algal blooms [1]. Sediments play a key role in regulating P availability in the water column in near shore environments by acting as an internal source and permanent sink for P. While P dynamics in sediments with high inputs of reactive organic matter have been reasonably well studied [2]–[7], relatively little is known about the diagenesis of P in oligotrophic coastal, low salinity settings.

The cycling of P in Bothnian Sea sediments is of specific interest given that recent mass balance models [8], [9] suggest that the sediments act as a major burial sink for P from the water column of the Baltic Proper, which is an adjacent strongly eutrophic basin [1], [10]. The total retention of P in the sediments in the Bothnian Sea has been estimated to range from 9.6×10^3^ ton yr^−1^ [8] to 18.6×10^3^ ton yr^−1^ [9]. Various processes may contribute to this burial of P. These include burial of P bound to Fe-oxides and in organic matter as well as sink-switching of the P from these phases to authigenic Ca-P forms [3], [11]–[14] or to vivianite (Fe_3_(PO_4_)_2_^.^8H_2_O) [15], [16]. While authigenic Ca-P is known to be a major burial sink for P in the ocean [17], the quantitative importance of vivianite burial remains to be determined. Based on geochemical data for Zambezi deep-sea fan sediments, März et al. [18] suggest that conditions for vivianite formation in marine sediments may be especially favourable below the sulfate-methane transition (SMT). These authors showed that upon sulfate depletion, resulting from anaerobic oxidation of methane (AOM), Fe^2+^ may accumulate in the porewater and no longer precipitate as Fe sulfide. When dissolved PO_4_ concentrations in the porewater are also high, supersaturation with respect to vivianite may be reached [18]. At present, little is known about the role of anaerobic oxidation of methane in facilitating vivianite formation in estuarine systems where the zone of AOM is located close to the sediment-water interface.

With a typical bottom water salinity of 5 to 6, the Bothnian Sea is a classical example of an oligotrophic, low-salinity coastal basin. While primary production in the system is low compared to the Baltic Proper [19], sediment organic carbon accumulation in the basin is still significant, because of major terrestrial input of organic matter from rivers [20], [21], [22]. Nitrate penetration in the sediment is limited to the upper cms due to active denitrification [20], [23]. Various studies of the geochemistry of the surface sediments in the Bothnian Sea in the 1980’s have shown that ferromanganese oxide layers and concretions [24], [25] are abundant. Given the ability of Fe-oxides to sequester large amounts of P [26], Fe-bound P could be a dominant sediment P phase in the Bothnian Sea. To our knowledge, no further information on the biogeochemistry of the surface sediments in the Bothnian Sea is available.

In this study, we use pore water and sediment data for 6 sites from contrasting sedimentary regimes in the Bothnian Sea to quantify the various burial phases of P and their potential role as a permanent sink for P at the system scale. We also assess the role of the depth of the sulfate-methane transition (SMT) in controlling sedimentary P dynamics. Our results show that Fe-bound P is the dominant form of P in sediments of the Bothnian Sea with sink-switching from Fe(III)-P to Fe(II)-P occurring below the zone of AOM at deep basin sites.

## Materials and Methods

### Ethics Statement

All necessary permits were obtained for the described field studies. RV Skagerak and RV Aranda had permission to sample the study sites for scientific purposes (granted by the Swedish and Finnish coast guard).

### Study Sites

**Figure 1:**
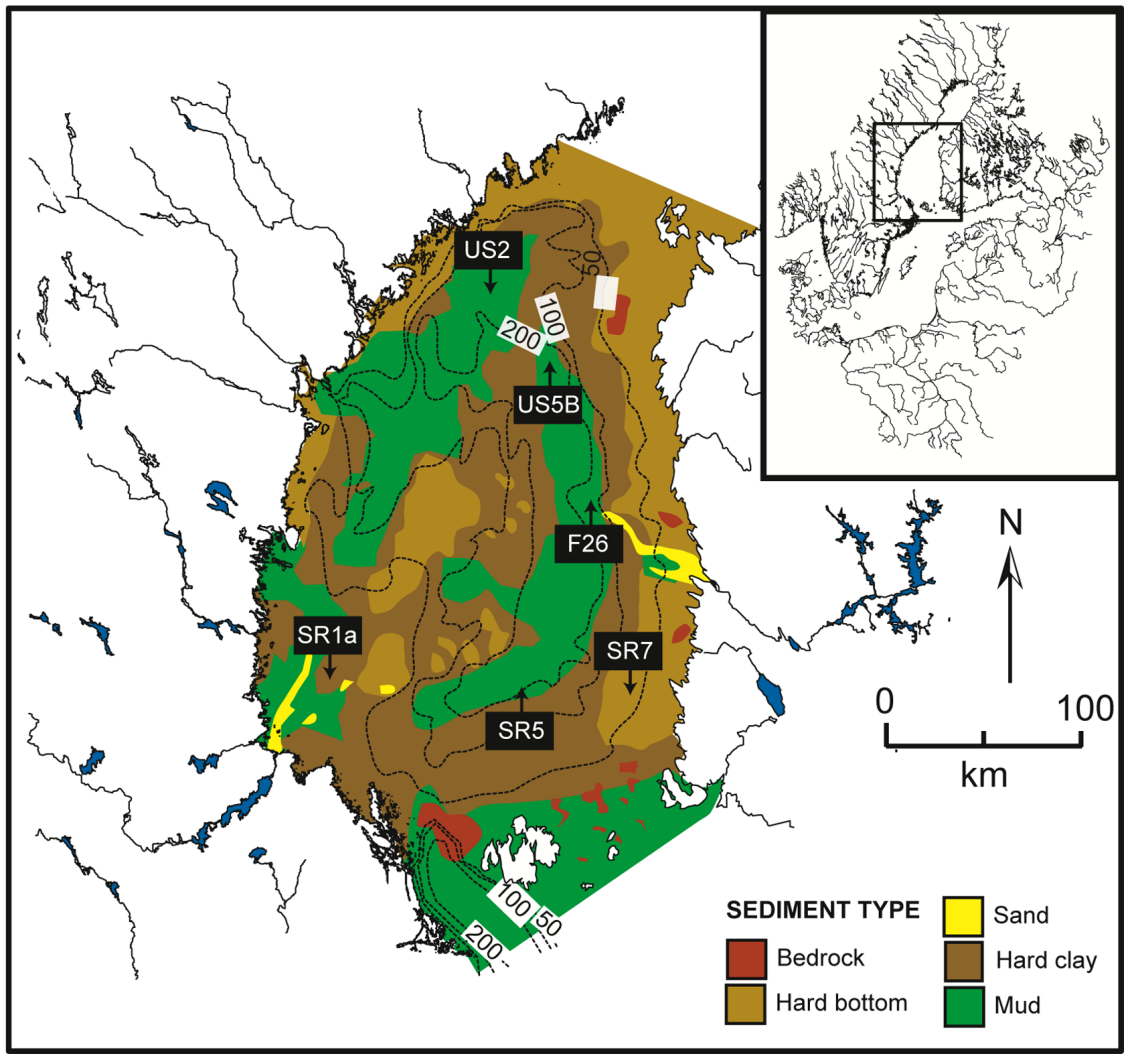
Location map of the study sites in the Bothnian Sea. Site characteristics are given in Table 1. Simplified bathymetric overlay and bottom sediment type are adapted from [73] and [72], respectively.

Six locations in the Bothnian Sea were sampled during research cruises with RV Skagerak in October 2008 and RV Aranda in June 2009 (Figure 1). The locations were separated into three groups based on water depth and average sedimentation rates (Table 1). Sediments at all sites were fine-grained. Sediment accumulation rates (SAR in g m^−2^ yr^−1^) and the annually accumulated layer of sediment at 10 cm (AAL in cm yr^−1^), were taken from the literature [27] and refer to the period prior to 2003. Group 1 sites (US2 and US5B) are located in the deepest part of the Bothnian Sea (204 m and 214 m, respectively) and are characterized by high rates of annual accumulation of sediment (1.6 and 1.5 cm yr^−1^, respectively). Group 2 sites (SR5 and F26) are located at intermediate water depths (124 m and 137 m, respectively) and are characterized by an AAL which is ∼5 times lower than in the deep basin. Finally, sites in Group 3 (SR7 and SR1a) are located at relatively shallow water depths (77 and 61 m, respectively) in areas with limited net sediment deposition. Five sites (all stations except US2) were sampled in October 2008. In June 2009, additional samples were taken at all group 1 and 2 sites (US2, US5B, SR5 and F26; Table 1).

**Table 1:** Characteristics of the 6 study sites based on conditions at the time of sampling in October 2008 and June 2009.

Site	Position & Timeof Sampling	O_2_ pen.(mm)	Sali-nity	Temp(°C)	Water depth (m)	SAR(g m^−2^ yr^−1^)	AAL(cm yr^−1^)	Group
**US2**	2009	∼40	6.2	4.2	204	5250	1.60	1
	62°50.72′ N							
	18°53.32′ E							
**US5B**	2008 & 2009	2 & 2	6.3 &	3.6 &	214	4090	1.49	
	62°35.17′ N		6.3	4.3				
	19°58.13′ E							
**SR5**	2008 & 2009	8.5 &	6.7 &	4.7 &	124	1160	0.32	2
	61°05.00′ N	17	6.4	3.5				
	19°34.78′ E							
**F26**	2008 & 2009	8.5 &	6.5 &	3.8 &	137	1200	0.33	
	61°59.02′ N	8	6.4	4.3				
	19°34.78′ E							
**SR7**	2008	7.5	5.9	3.9	77	270	0.04	3
	61°05.01′ N							
	20°35.79′ E							
**SR1a**	2008	8.5	5.4	4.7	61	930	0.09	
	61°14.00′ N							
	17°39.79′ E							

O_2_-pen.: oxygen penetration into the sediment as measured with micro-electrodes. Temp: temperature of the bottom water. Sediment accumulation rates (SAR) and the annually accumulated layer of sediment (AAL in cm/yr, at a depth of 10 cm) are based on ^137^Cs dating and sediment porosity and density as described in the study of Mattila et al. [27].

### Bottom Water and Pore Water Analyses

Bottom water oxygen, temperature and salinity were measured with a CTD system to which an oxygen sensor was attached. Sediment cores (20–40 cm of sediment and at least 10 cm of overlying water) were recovered using a multi-corer (10 cm i.d.). At each site, one core was immediately sliced in a N_2_-purged glove box at in-situ temperature. A small section of each slice was stored anoxically at 4°C for sediment analyses. Pore water was collected either by centrifugation of the remaining sediment for 10 to 30 minutes at 2500g (2008: US5B, SR5; 2009: US2, US5B, SR5) or by suction using rhizons [28] placed in an additional sediment core from the same multi-core cast (2008: SR5. F26, SR7, SR1a; 2009: US2, US5B, SR5, F26). Pore waters were sub-sampled under N_2_ for analysis of dissolved PO_4_, NH_4_^+^, S, Fe, Mn and SO_4_^2−^. Sub-samples for PO_4_ were acidified with concentrated HCl and were stored at 4°C until analysis. Sub-samples for NH_4_^+^ were stored frozen. Both PO_4_ and NH_4_^+^ were determined colorimetrically on a nutrient auto-analyzer (Bran and Luebbe). Sub-samples for total dissolved S, Fe and Mn were acidified with HNO_3_ (2008) or HCl (2009) and stored at 4°C until analysis by ICP-OES. Dissolved S is expected to represent SO_4_^2−^, because dissolved sulfide is assumed to be released during the acidification [29]. Subsamples for SO_4_^2−^ were stored at 4°C until analysis with ion chromatography (IC; Dionex). Pore water SO_4_^2−^ from ICP-OES and IC-analyses generally compared well, but there was significant scatter in the IC-data. This was mostly observed at higher SO_4_^2−^ concentrations, likely due to interference with other (unknown) components in the pore water. As an example, the results for site US5B in October 2008 and June 2009 are shown in Figure S1 in File S1. Because of the more consistent results, we subsequently present only SO_4_^2−^ from ICP-OES. Pore water profiles for rhizons and centrifuged samples were similar. Where available, we present pore water data from centrifuged samples, because of the higher depth resolution. Dissolved oxygen microprofiles were obtained on board using a Unisense oxygen sensor attached to a micromanipulator. Methane concentrations in pore water were determined at stations US2, US5B and F26 in June 2009. Sediment samples were taken from a multi-core with pre-drilled sample ports using a 20 ml syringe directly upon core retrieval. Samples were equilibrated with a saturated NaCl solution in sealed glass vials (65 ml) closed with a rubber stopper and screw cap. A headspace of 10 ml nitrogen was inserted and methane concentrations in the headspace were determined with a Thermo Finnigan Trace GC gas chromatograph (Flame Ionization Detector).

### Sediment Analyses

Sediment samples were freeze-dried, powdered in an agate mortar in a glove box and split into oxic and anoxic fractions. Sediment porosity was calculated from the weight loss upon freeze-drying. Oxic samples were used for total elemental and organic C and N analyses. The total elemental content of the sediments was determined using a HF-ClO_4_-HNO_3_ destruction followed by analysis with ICP-OES. Sediment total C and N were measured using an elemental analyser (Fison Instruments model NA 1500 NCS). All N was assumed to be in organic form. Organic C was (Corg) estimated by subtracting CaCO_3_-associated C from total C. CaCO_3_-associated C was estimated from total Ca after correcting for Ca in clays using total Al contents and a Ca/Al weight ratio of 0.071 [30]. The average Corg content in surface sediments for group 1 and 2 sites is 2.7 wt% which is similar to the average value for the region reported earlier [22].

Anoxic samples were used for sediment P speciation to avoid artefacts linked to pyrite oxidation during sample handling and storage [31]. Sediment P was fractionated into exchangeable P, Fe-bound P, authigenic Ca-P (this includes carbonate fluorapatite (CFA), biogenic Ca-P and CaCO_3_-bound P), organic P and detrital Ca-P using the SEDEX procedure [32] with omission of several of the wash steps [11]. Reactive P is defined as the sum of exchangeable P, Fe-bound P, authigenic Ca-P and organic P. Sediments were shielded from oxygen until step 3 of the SEDEX procedure. The Fe extracted in step 2 of the SEDEX procedure is termed CDB-Fe, where CDB stands for citrate-dithionite-bicarbonate. CDB-Fe is often used as a measure of total Fe-oxides in the sediment. However, vivianite [33] and some pyrite [26] are also extracted in CDB solutions as used in step 2 of the SEDEX procedure, even when sediments are shielded from the atmosphere. Thus, vivianite Fe and P and a part of the pyrite Fe are included in the CDB-Fe and P contents. To assess the potential role of amorphous Fe-oxides for P binding, ascorbate-extractable P and Fe [34] were determined for a selection of 7 and 8 samples from cores US5B and SR5, respectively.

### Diffusive Fluxes of Methane and Sulfate

Diffusive fluxes of methane and sulfate into the sulfate-methane transition (SMT) were calculated from pore water gradients using Fick’s first law [35]:
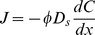
(1)where J is the diffusive flux in mmol m^2^ yr^−1^, φ is the measured porosity, D_s_ is the sediment diffusion coefficient (m^2^ yr^−1^), C is the concentration of either sulfate or methane (mM), and x is the depth (m). Sediment diffusion coefficients were calculated following:
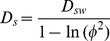
(2)where Dsw was corrected for in-situ temperature and salinity [36].

## Results

**Figure 2:**
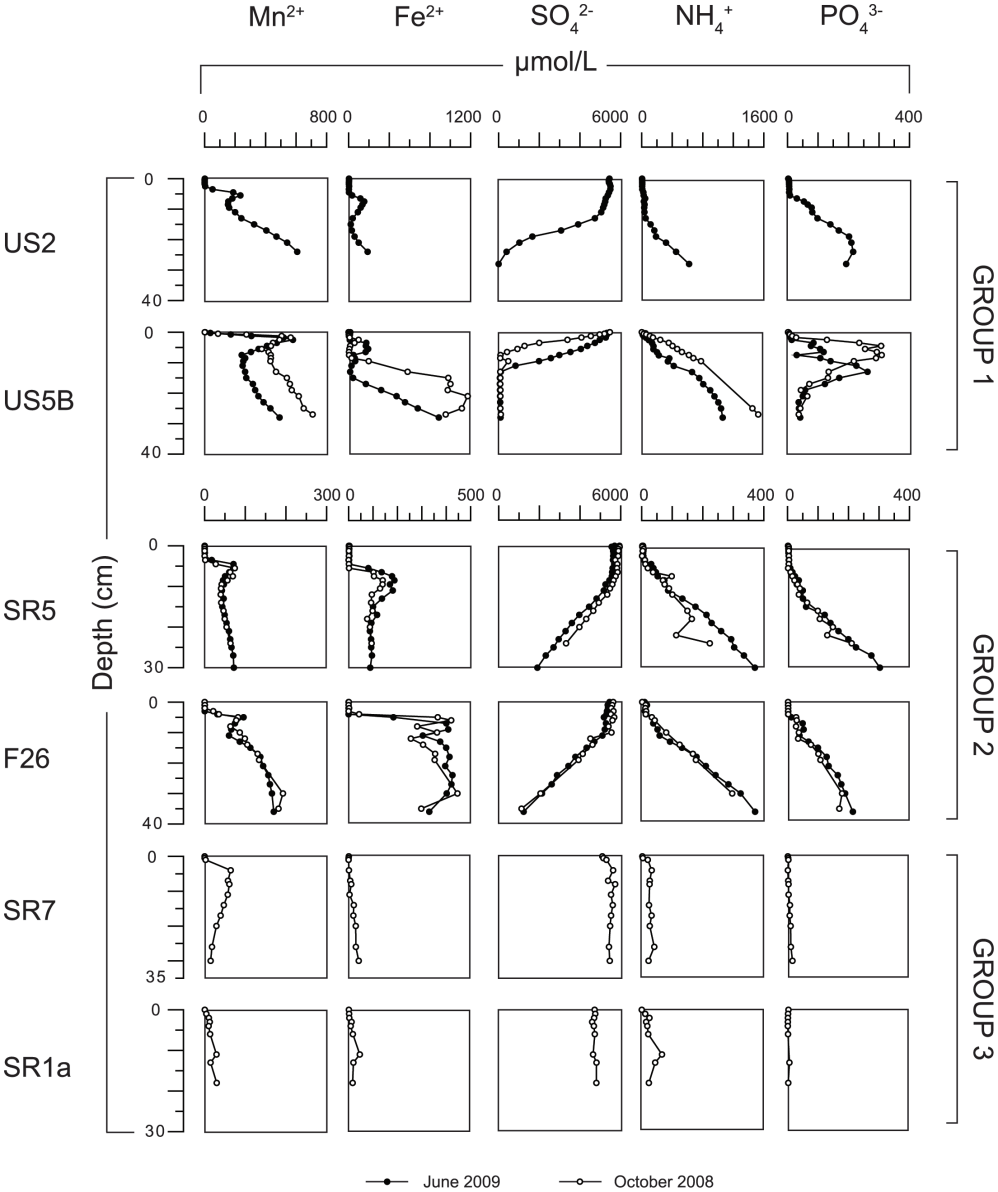
Porewater profiles of key components for June 2009 and October 2008. Units are in µmol/l. Note the different scales for group 1 and groups 2 and 3.

Bottom waters and surface sediments at all sites were oxic with oxygen penetration in the sediment varying from 2 mm to several cms (Table 1). Bottom water salinity and temperature were low and relatively constant at all sites (average values of ca. 6 and 4°C, respectively; Table 1). Pore water and sediment data are provided in a supplementary data file (Tables S1 to S6 in File S1). Pore water profiles of the group 1 and 2 sites mostly display the typical signature of depositional locations with an increase in dissolved Mn^2+^, Fe^2+^, NH_4_^+^ and PO_4_ and a decrease in dissolved SO_4_^2−^ with depth below the oxic surface layer (Figure 2). Gradients in pore water NH_4_^+^ and SO_4_^2−^ profiles at group 1 (US2 and US5B) sites are significantly higher when compared to those of group 2 (SR5 and F26). Dissolved PO_4_ concentrations at all group 1 and 2 sites are of a similar order of magnitude as those of NH_4_^+^ and generally increase with depth in the sediment. The only exception is US5B, where PO_4_ concentrations decline strongly when pore water SO_4_^2−^ is depleted. At this station, maxima in dissolved Fe^2+^, Mn^2+^, NH_4_^+^ and PO_4_ and the depth of SO_4_^2−^ depletion are all located at slightly greater depths in June 2009 when compared to October 2008. At the shallower group 2 sites, no such change in pore water profiles between sampling campaigns is observed. At group 3 sites (SR7 and SR1a), pore water concentrations of all constituents are low and show only relatively minor change with depth.

**Figure 3:**
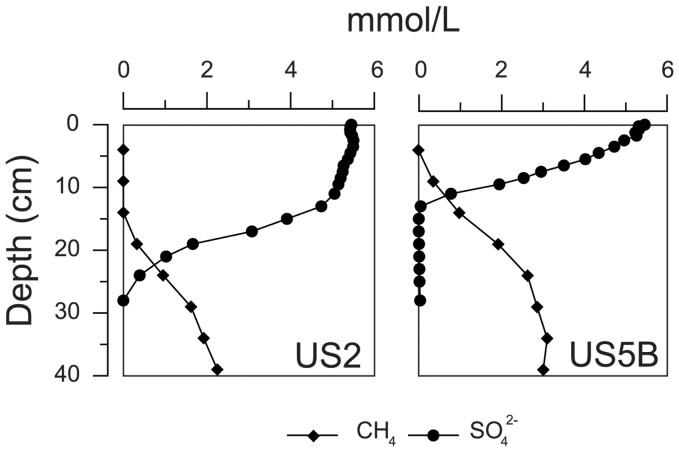
Porewater profiles of SO_4_^2−^ and CH4 for sites US2 and US5b for June 2009.

Where downward diffusing SO_4_^2−^ meets upward diffusing CH_4_ at sites US2 and US5B (group 1), both components are removed from the pore water, suggesting anaerobic oxidation of CH_4_ with sulfate (Figure 3). Corresponding diffusive fluxes of sulfate and methane into the SMT in June 2009 are 0.67 and 0.36 mmol m^−2^ d^−1^ for site US2 and 2.2 and 0.93 mmol m^−2^ d^−1^ for site US5B, respectively. These fluxes suggest that ∼50% of sulfate removal is associated with AOM with the remainder being coupled to organic matter degradation. Note, however, that CH_4_ concentrations are higher than saturation levels at atmospheric pressure (∼2 mM) [37]. This indicates that degassing of some of the methane upon core retrieval likely occurred [38]. No CH_4_ was detectable in the pore water at site F26 (group 2).

**Figure 4:**
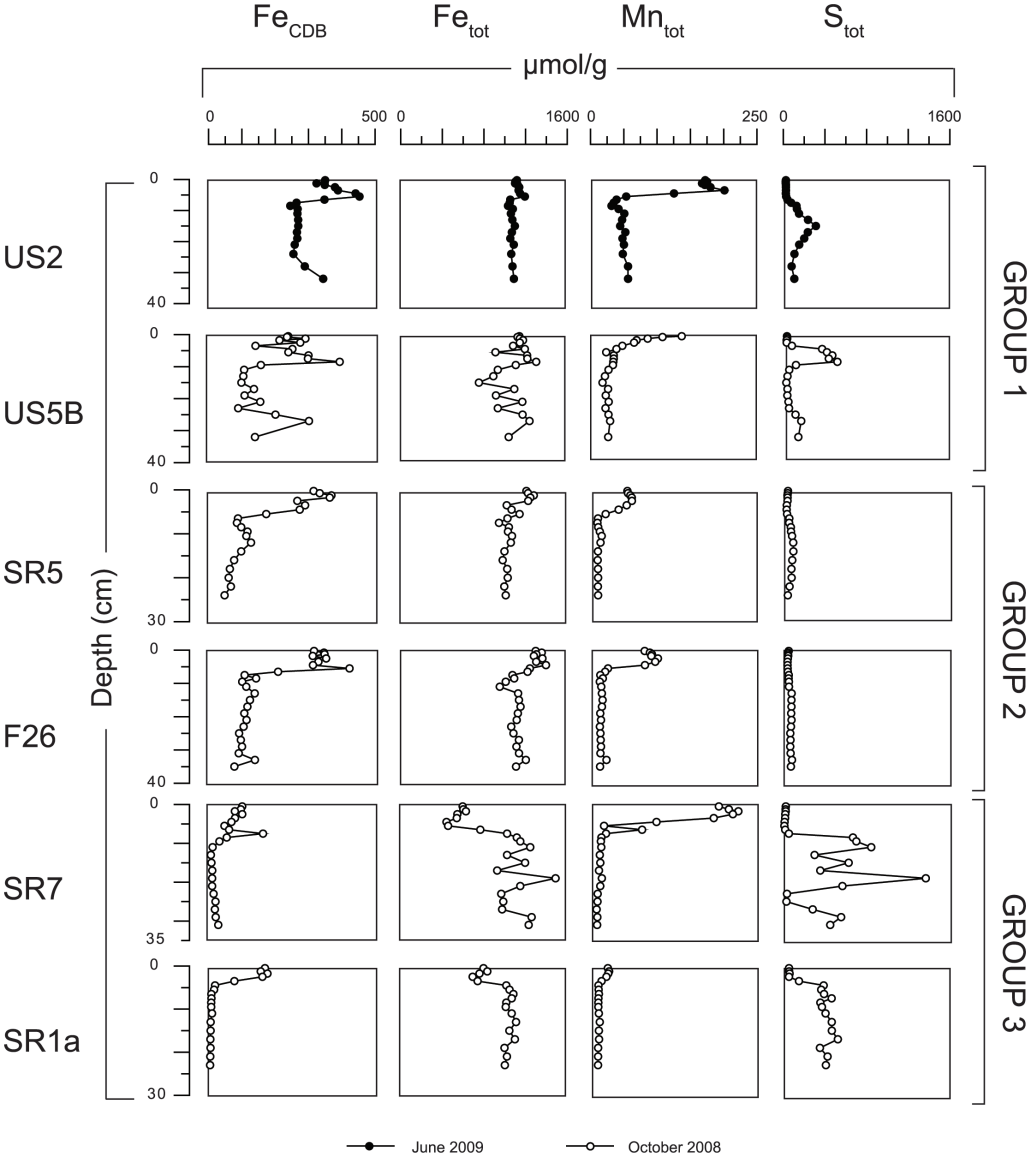
Sediment depth profiles of CDB Fe, total Fe, total Mn and total S (in µmol/g). CDB-Fe is typically used as a measure of total Fe-oxide Fe in the sediment but may include vivianite-Fe [33] and Fe-sulfides [26].

All sites are enriched in CDB-Fe and total Mn in the surface sediment, suggesting the presence of Fe- and Mn-oxides (Figure 4). At group 1 and 2 sites, surface enrichments in Fe are also visible in the total Fe profile. At these locations, CDB-Fe persists below the oxic surface layer over the full length of the sampled cores. Sulfur is present at depth in the sediment at all sites. A distinct maximum in total S and Fe is observed at the SMT at site US5B (Figure 3 and 4), whereas a much broader maximum in total S that tails upward and downward is found at US2 (Figure 4).

**Figure 5:**
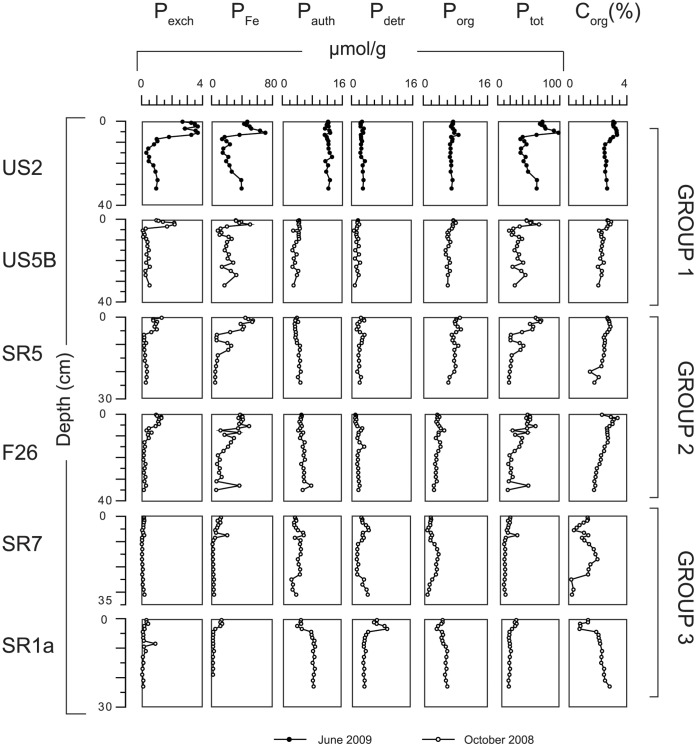
Sediment phosphorus speciation (in µmol/g) and organic carbon contents (C_org_ in wt%) for all sites.

**Figure 6:**
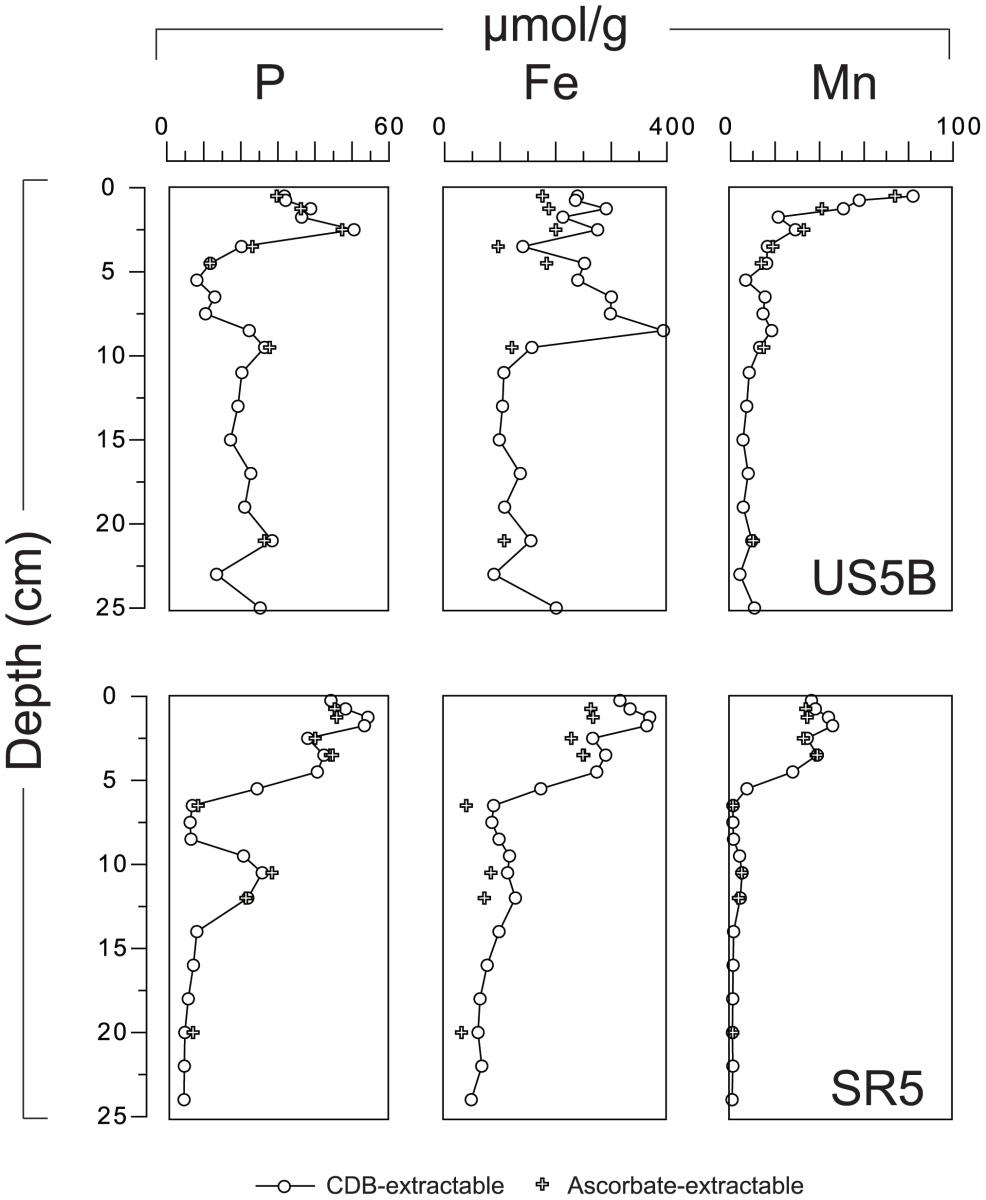
Depth profiles of sediment P, Fe and Mn (in µmol/g). Sediments for sites US5B and SR5 were extracted with CDB (filled circles) and ascorbate (open circles).

Total P contents range from ∼20 to 100 µmol g^−1^ (Figure 5). All sites show surface enrichments of exchangeable and Fe-bound P where Fe-bound P accounts for up to 80% of total P. The Fe-bound P is an important burial sink at group 1 sites and at one of the group 2 sites (F26). Authigenic Ca-P contents are mostly constant with depth and range up to ca. 12 µmol g^−1^ at sites SR1a and US2. At the other sites contents are lower and vary between 3 and 6 µmol g^−1^. Detrital P is generally a minor phase and shows relatively little variation with depth. Organic P (P_org_) accounts for on average 20% of total P burial with depth profiles varying considerably from site-to-site. Organic carbon (C_org_) contents are high at all sites and range mostly from 1.3 to 3.3 wt%. Depth profiles of P and Mn extracted with CDB and ascorbate (sites US5B and SR5) are similar (Figure 6). On average, ca. 50 µmol/g more Fe is extracted with CDB than with ascorbate.

## Discussion

### Sources of Organic Matter and Trends in Mineralization Rates

While productivity in the Bothnian Sea is relatively low when compared to other areas of the Baltic Sea [19], changes in water transparency suggest that phytoplankton biomass in the area has increased over the past century, with the changes being caused by eutrophication [39]. As a consequence, the input of locally produced organic matter to the sediments is expected to have increased with time. However, terrestrial sources are the most important and account for more than 65% of dissolved and particulate organic matter in the Bothnian Sea [40], [41]. Land uplift for the whole Gulf of Bothnia (i.e. including the Bothnian Bay) is 7 mm per year. This uplift leads to significant erosion and lateral transport of material from shallow areas into the deep basins of the Bothnian Sea [42]. A major proportion of the organic matter is thus relatively refractory. These processes explain the relatively high contents of organic carbon in the sediments of this oligotrophic basin and the observed increase with water depth.

Pore water NH_4_^+^ profiles point towards considerable mineralization of the deposited organic matter at group 1 and 2 sites. Concentrations of NH_4_ increase with sedimentation rate and thus with water depth in the basin (Table 1; Figure 2), indicating that rates of organic matter mineralization also increase with water depth, e.g. [35]. Macrofauna have been shown to actively bioturbate the sediments at sites US2 and SR5 in the Bothnian Sea [43]. Bioturbation is more limited at site US5B due to the absence of deep-burrowing worms (J. Norkko, personal communication). Given the high contents of Fe- and Mn oxides in the surface sediment, the resulting downward transport of particles is expected to lead to a significant contribution of dissimilatory Fe and Mn oxide reduction to organic matter degradation at group 1 and 2 sites. Trends in pore water SO_4_^2−^ are in line with our grouping of sites and corroborate that mineralization rates are highest at the deep basin sites of group 1. Solid phase S profiles (Figure 4) suggest that sulfate reduction leads to scavenging of dissolved Fe^2+^ from the pore water and formation of Fe-sulfides at all sites.

Seasonal changes in input of organic matter to the sediment are likely not very important in this oligotrophic basin where a major proportion of the organic matter is terrestrial [40], [41]. This is supported by the lack of change in the pore water profiles for the group 2 sites from October 2008 to June 2009. At the deeper group 1 site US5B, differences between the 2 sampling times are observed, however. This may be indicative of a small downward shift of the SMT from 2008 to 2009 (on a scale of several cm’s) or, alternatively, may reflect spatial variability at the site.

### Anaerobic Oxidation of Methane (AOM) with Sulfate

Pore water profiles of CH_4_ and SO_4_^2−^ suggest that anaerobic oxidation of methane (AOM) coupled to sulfate reduction is occurring in the sampled sediment intervals at both deep basin sites (group 1, US2 and US5B). The SMT extends over an interval of ca. 10 to 15 cm. Such a relatively narrow SMT is in line with earlier work on AOM in coastal sediments [44]–[46]. Our calculated diffusive methane fluxes into the SMT (∼0.4 to 0.9 mmol m^−2^ d^−1^) are within the range of AOM rates typically observed for coastal sites (0.17–1.5 mmol m^−2^ d^−1^) [44], [47], [48]. AOM coupled to sulfate reduction leads to a production of sulfide that will reductively dissolve Fe-oxides and react with dissolved Fe^2+^ to form Fe-sulfides. Maxima in total S in this zone are assumed to be indicative of accumulation of such Fe-sulfides.

Similar Fe-sulfide maxima have been observed at the SMT in various long-core records, for example in sediments of the Amazon deep sea fan [49], Zambezi deep sea fan [18] and western Argentine Basin [50]. Such enrichments are the result of non-steady state diagenesis and, in these earlier studies, have been recognized as such. The authors attribute the enrichments they observe to changes in sedimentation rates and organic matter input followed by fixation of the SMT at a given sediment level several meters below the seafloor for an extended period of time (up to thousands of years).

Distinct maxima in Fe sulfide cannot form in anoxic sediments under steady state conditions. Instead, sulfate reduction will lead to a gradual increase in the sediment content of Fe-sulfides with depth. This increase will continue until either sulfate or Fe(III) is depleted. Because there is no removal of Fe sulfide in anoxic sediment, once it is formed, the Fe sulfide content will stay at a constant value and show no further change with depth [35], [51]. Such depth trends in FeS and FeS_2_ profiles have been observed in many coastal sediments and have been quantitatively analysed using various steady state and dynamic reactive transport models [52], [53], [54].

The enrichment of authigenic Fe-sulfides at both group 1 sites (US2 and US5B) is thus indicative of a non-steady state phenomenon. However, in contrast to the earlier studies for deepsea cores mentioned above, the enrichment in Fe sulfide is located close to the sediment-water interface (upper 20 cm) in rapidly accumulating sediment (∼1.5 cm yr^−1^). Given that a downward shift of the SMT is not possible in a setting where bottom waters have been continuously oxic over the past decades, the only alternative is an upward shift of the SMT. The most pronounced peak in sulfur is found at US5B and the size of this peak can provide some insight in the relevant time scale. With an average diffusive influx of sulfate of ∼1 mol m^−2^ y^−1^ (based on the pore water profile for 2008; Figure 2), and an S content of ∼7 mol m^−2^ for the enrichment (from the data in Figure 4), only ∼7 years would be required to form this layer. This indicates that the upward shift likely took place within the last 10 years. The broad maximum in sediment S for US2 suggests significant temporal variability in the depth of the SMT at this site and/or possibly vertical expansion of the peak due to bioturbation. The more distinct maximum in total S at site US5B suggests that here the position of the SMT has moved up more abruptly and has varied over a narrower depth range over the past decade and is not significantly affected by bioturbation (Figures 3 and 4).

At group 2 sites, only very minor enrichments in solid S are observed. Here, the SMT is located below our sampled depth interval. These were likely the conditions at sites US2 and US5B prior to the upward shift in the SMT. Group 3 sites are characterized by surprisingly high total S contents below 10 cm when taking into account the very low sedimentation rates and low rates of organic matter mineralization. This may be the result of long-term accumulation of Fe sulfide at a very low rate or may be the remnant of another diagenetic regime.

### Phosphorus Dynamics and the Link with AOM

Pore water PO_4_ concentrations at many sites are of the same order of magnitude as those of NH_4_^+^ (Figure 2). If organic matter degradation were the major source of PO_4_, a ratio of N:P in pore water closer to the Redfield ratio of 16 would be expected. This suggests that most dissolved PO_4_ in the pore water is derived from reduction of Fe-oxides and release of associated P at depth in the sediment. A major proportion of this PO_4_ is trapped again upon upward transport into the oxygen and nitrate containing zone, either through sorption to Fe-oxides or co-precipitation with dissolved Fe^2+^. This recycling of Fe and P in the sediment is well described for other near coastal and offshore marine systems [2], [11], [55]. At our study sites, this has allowed major surface enrichments of Fe-oxides and associated P to form, which account for up to 80% of the total P in these sediment layers.

Authigenic Ca-P at most sites is relatively constant with depth and values are mostly low. These near vertical profiles suggest that this material is not formed in-situ. Slightly higher contents are observed at sites US2 and SR1a. At SR1a, the abrupt rise in authigenic Ca-P at ∼10 cm depth is matched by a similar change in total Fe, P, total S and organic C suggesting variations in bulk sediment composition are responsible and not authigenesis. We conclude that sink-switching of organic or Fe-bound P to authigenic Ca-P, as is often observed in continental margin settings [3], [11], is limited in Bothnian Sea sediments. Similar results were found for 5 out of 6 locations in a recent study of P burial in the central and southern Baltic Sea [6]. Explanations include possible undersaturation with respect to a Ca-P precursor phase, as suggested based on long-term experiments on apatite formation in Baltic Sea water [56] or a lack of polyphosphate from e.g. algae in the sediment, thus precluding the transformation of polyphosphate to authigenic apatite as observed in other coastal sediments [57].

**Figure 7:**
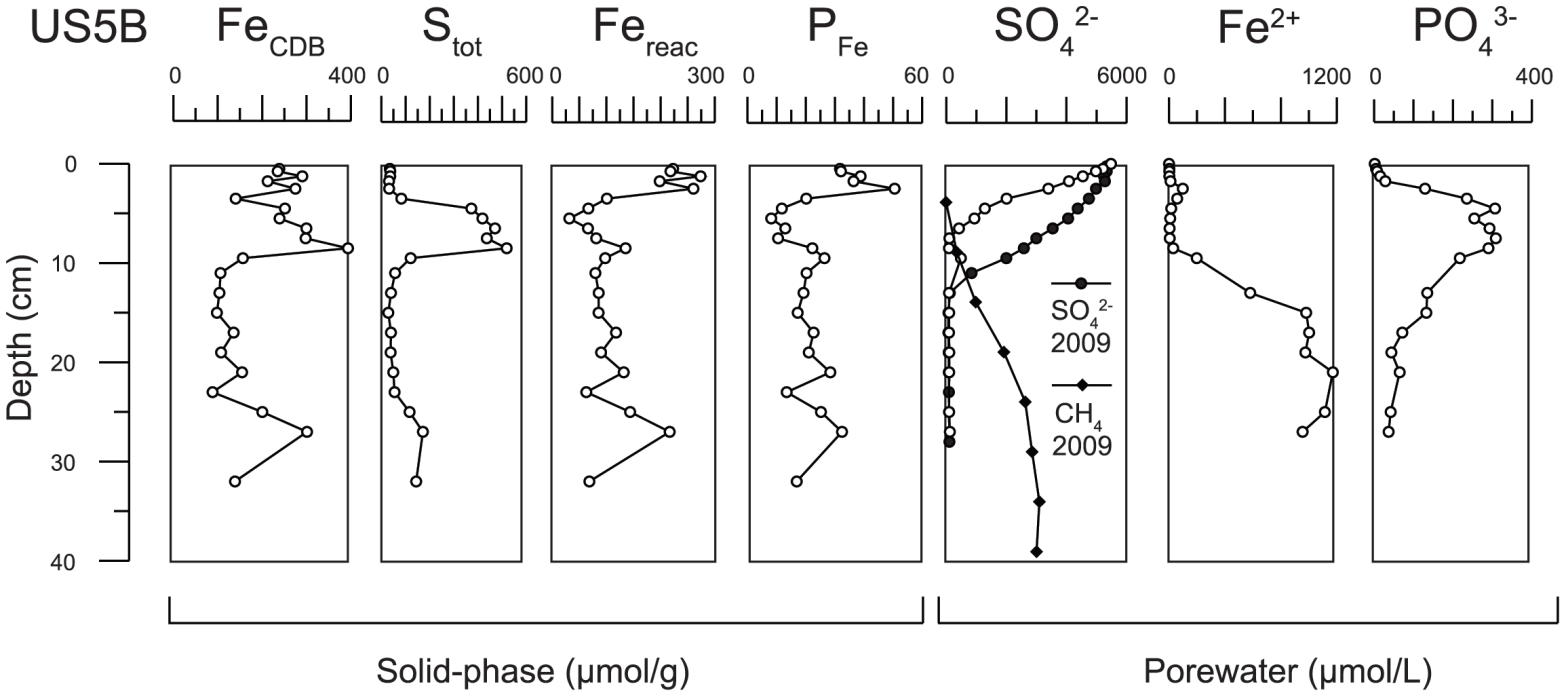
Compilation of key sediment and porewater profiles for US5B. Depth profiles of sediment contents of CDB-Fe, total sulfur (S_tot_), reactive Fe oxides (Fe_reac_; see text) and Fe-bound P in units of µmol/g, and depth profiles of porewater concentrations of methane, sulfate and dissolved Fe^2+^ and PO_4_ (in µmol/l) for site US5B. Data are for October 2008, unless indicated otherwise.

At group 1 and 2 sites, Fe-bound P acts as the major burial sink for P (Figure 5). Contents of CDB- and ascorbate P (Figure 6) are similar indicating that preservation of crystalline Fe oxides and associated P plays a negligible role in this Fe-bound P burial. At sites US2 and US5B, it is particularly striking that Fe-bound P increases again below ca. 10 cm depth after an initial decline. This suggests the formation of an Fe(II)-P phase, such as vivianite (Fe_3_(PO_4_)_2_^.^8H_2_O) at depth. Here, we will concentrate on site US5B, because for this location the trends in the pore water below the SMT are also captured. To estimate the amount of Fe that is potentially associated with P, we first correct CDB-Fe for the Fe associated with Fe-sulfides that may be dissolved during this extraction [5] and, as a first approximation, we assume that all sulfur is present in the form of pyrite (Figure 7). The resulting profile of remaining, non-sulfidized reactive Fe (Fe_reac_) is similar to the Fe-bound P profile at US5B: both profiles show minimum values at depths where S contents show a maximum. This is in accordance with reductive dissolution of Fe oxides and release of associated P in the SMT and precipitation of both Fe and PO_4_ below the SMT. The pore water profile of dissolved PO_4_ confirms the release of P from Fe-oxides in the SMT and precipitation of PO_4_ when Fe^2+^ appears in the pore water below this zone (Figure 7).

Strikingly similar Fe^2+^ and PO_4_ profiles in the zone of AOM were recently reported by Sivan et al. [58] for lake sediments and were attributed to vivianite formation. Sink-switching from Fe-oxide bound to Fe(II)-P phases has been demonstrated earlier for lake sediments [59], [60], but is not well-documented for estuarine environments [17]. Apart from suboxic Amazon fan sediments where vivianite nodules were found [16], [61], the only evidence for vivianite formation in surface sediments of near shore coastal settings is based on the saturation state of the pore waters [15]. We also calculated the saturation indices for vivianite for the pore water for all Bothnian sites using PHREEQC with the LLNL database [62] and the solubility constant from Al-Borno and Tomson [63]. Our results indicate undersaturation at group 3 sites (SR7 and SR1a) but oversaturation below a depth of ∼5 cm at group 1 and 2 sites (Tables S5 to S7 in File S1). Saturation indices are highest at group 1 sites, i.e. at US2 and US5B, confirming that conditions at these deep basin sites are most conducive for vivianite formation.

**Figure 8:**
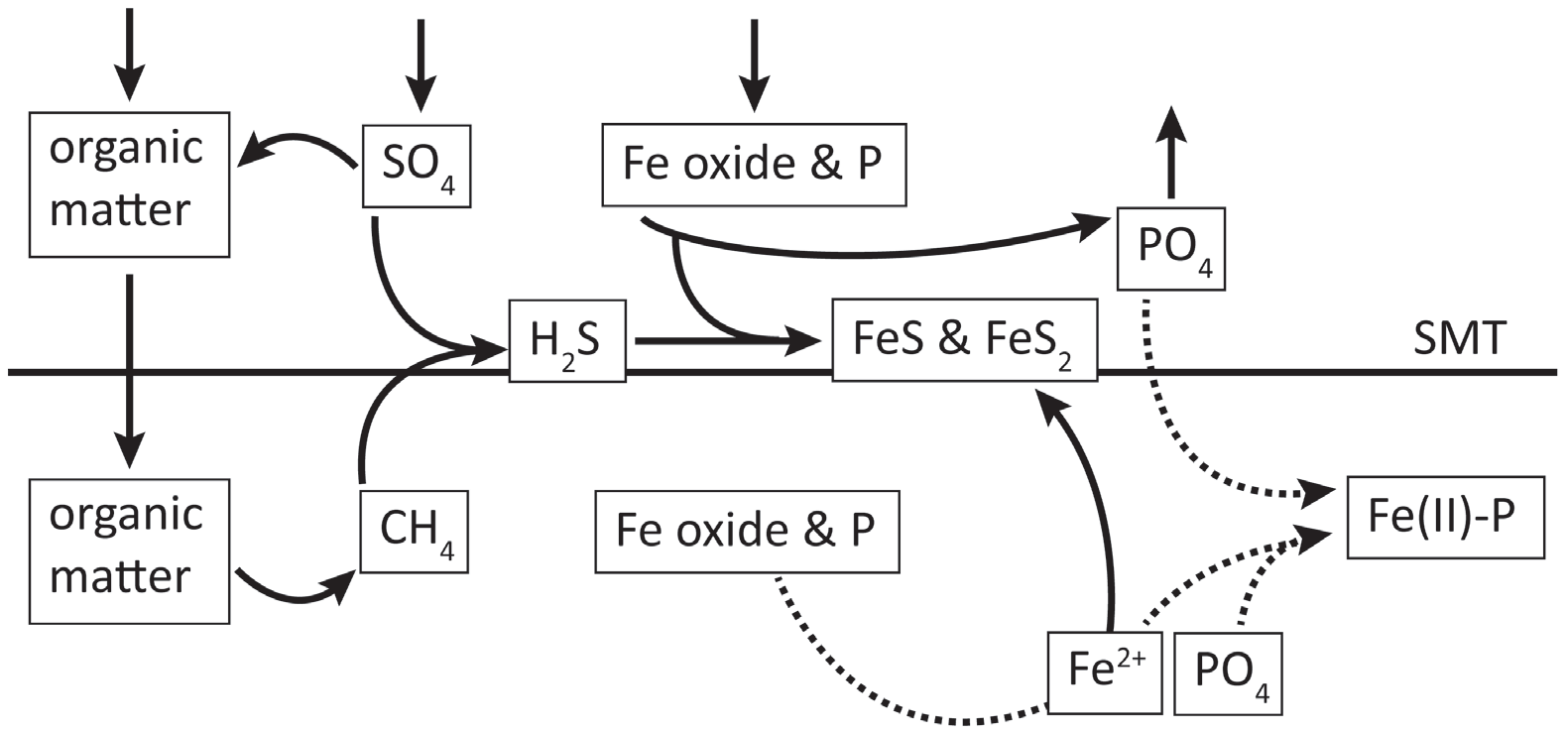
Schematic of key processes affecting S, Fe, CH_4_ and P in and below the SMT. The dotted lines indicate how the release of Fe^2+^ and PO_4_ from Fe-oxides lead to Fe(II)-P formation.

The mechanism that we postulate for the present-day formation of Fe(II)-P in the deep basin sediments of the Bothnian Sea is summarized in Figure 8. An increased input of organic matter to this area starting at least several decades ago [64] likely initiated increased rates of methanogenesis in these sediments. Combined with increased rates of sulfate reduction, possibly also associated with a more recent increase input of organic matter, this likely led to the observed upward shift of the SMT. Anaerobic oxidation of methane removes a considerable amount of the sulfate that is diffusing into the sediment from the overlying water. The sulfide that is generated reductively dissolves sediment Fe oxides, with the reduced Fe and S being sequestered as pyrite and FeS. Part of the PO_4_ released from the Fe oxides upon their dissolution in this subsurface layer diffuses upwards and is trapped in the surface sediment as Fe-oxide bound P or escapes to the overlying water. A major part of the PO_4_ diffuses downwards, however, where it meets upward diffusing Fe^2+^ and precipitates as Fe(II)-P.

Dissolved Fe^2+^ and Mn^2+^ concentrations at depth in the sediment are extremely high (>1 mM and 0.7 mM, respectively). Reductive dissolution of metal oxides by sulfides is unlikely to be responsible given that (1) there is no sulfate below 10–15 cm depth that could be reduced to form sulfide; (2) the sulfide generated at the SMT directly encounters dissolved Fe^2+^ and reactive Fe(III) and thus will not be able to diffuse downwards into the zone where most of the Fe reduction takes place. The abrupt transition between sulfide and Fe^2+^ containing pore waters that we propose here is also what is observed below the SMT in Black Sea sediments [65], [66] and coastal sediments of Aarhus Bay, Denmark [67]. There are two alternative mechanisms that can explain the high dissolved metal concentrations in the pore water. The first mechanism is dissimilatory Fe and Mn oxide reduction, i.e. the reduction of Fe and Mn oxide coupled to organic matter degradation. This process is energetically more favorable than sulfate reduction but may be kinetically hindered [58], [68] explaining the persistence of Fe-oxides in the zone of sulfate reduction. The second possible mechanism is the reaction of CH_4_ with Mn and Fe oxides [69]:
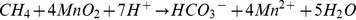
(3)

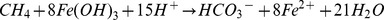
(4)


In the sediment at site USB, easily reducible Fe and Mn oxides are abundant in the zone where methane is present (Figure 7).

Regardless of the process driving the production of dissolved Fe^2+^, the high concentration at depth plays a key role for the sequestration of P in the sediment. If, for example, all Fe^2+^ would be sequestered with sulfide, no Fe(II)-P could be formed and all the PO_4_ released from the Fe-oxides would diffuse upwards. While some of the PO_4_ would be bound to existing Fe-oxides in the surface sediment, the lack of new formation of Fe-oxides would ultimately lead to a saturation of sorption sites and an increased release of dissolved PO_4_ to the overlying water. Thus, through its role in Fe-P retention, the Fe^2+^ release below the zone of AOM ultimately may control sediment-water exchange of PO_4_ at this site. The formation of sediment Fe(II)-P below the zone of AOM could potentially even act as a key feedback preventing further eutrophication of the Bothnian Sea.

The Fe/P ratio of the solids formed is of specific importance in this context. While the minimum stoichiometric ratio of Fe oxides/Fe-oxide bound P is 2, typical ratios in oxic marine surface sediments are higher at ∼10 [26]. Vivianite, has a stoichiometric ratio of 1.5 implying that sequestration of PO_4_ as Fe(II)-P requires less Fe. Because of the more limited requirement for Fe, increased sequestration of P as Fe(II)-P instead of Fe(III)-P reduces the impact of increased sulfate reduction and Fe sulfide formation on Fe-P retention in the sediment. Unfortunately, we can not accurately determine the Fe/P ratio of Fe-oxide-P and Fe(II)-P in the sediment. However, we do typically observe a stronger decline in Fe relative to P with depth in the sediment implying lower Fe/P ratios at greater depth.

Various studies of P dynamics in lake and marine sediments have emphasized the role of coupled Fe-S-P cycling for sediment P retention, where increased sulfate reduction leads to more P release to the water column [70], [71]. Our work shows that this model may have to be extended to include interactions with CH_4_. Further work is clearly needed to elucidate and quantify the coupled cycling of Fe, S, CH_4_ and P at these deep basin sites in the Bothnian Sea and the potential ecosystem impacts.

### Burial of P in the Bothnian Sea: Regional Importance

Mass balance models [8], [9] suggest that the Bothnian Sea is a major burial sink for P originating from the eutrophic Baltic Proper. Assuming that burial rates for our sites are representative for a larger region with a similar bathymetry and sediment composition, we can roughly estimate the total reactive P burial in the basin (Table 2). Estimates of areas for each depth range (60–99 m, 100–179 m, 179 m-maximum depth; corresponding to sites in groups 1, 2 and 3) were obtained using data from Al-Hamdani and Reker [72]. Burial rates for each site were estimated by multiplying sedimentation rates (Table 1) and reactive P contents (all P forms except detrital P) at 10 cm depth (Figure 5). At the chosen depth, most of the diagenetic enrichment of Fe-oxide bound P is not included in the estimate. Nevertheless, this is a rough approximation because it may still include some P that may be mobilized or P that is not truly reactive.

**Table 2:** Reactive P burial in the Bothnian Sea.

Depth range (m)	Area (km^2^)	Mud (%)	Site group	Reactive P burial(mmol m^−2^ yr^−1^)	Reactive P burial (ton yr^−1^)
179 m - bottom	1435	97	1	166	7166
100–179 m	11614	68	2	18	4368
60–99 m	21575	34	3	8	1773
				Total burial	13307

Burial estimates are based on depth ranges assigned to each site group and assuming burial in muddy areas only. Data on bathymetry and corresponding areas were taken from [73].

Despite the small area of the deep basins of group 1 (4% of the total area considered), they may account for an estimated 54% of the reactive P burial in the Bothnian Sea (Table 2). The depositional sites of group 2 at intermediate depths (33% of the total area) are estimated to account for 32%, whereas the shallow sites of group 1 (62% of the surface area) account for only 13% of the reactive P burial. The total burial of reactive P is estimated at 13,307 tons yr^−1^. This estimate lies within the range of 9,600 to 18,600 tons yr^−1^ suggested in earlier mass balance studies [8], [9] and confirms the important role of Bothnian Sea sediments in sequestering P. A significant proportion of this P is currently being sequestered as Fe-bound P.

### Conclusions

Our results show that Fe-bound P is an important sediment phase in the Bothnian Sea and acts as major burial sink for P. While P is associated mainly with Fe oxides in the surface sediment, we find strong indications that a reduced Fe-P phase, possibly vivianite (Fe_3_(PO_4_)_2_^.^ 8H_2_O), is formed below the sulfate-methane transition (SMT) at two of our study sites. We suggest that this Fe(II)-P mineral is an important P sink in sediments of the Bothnian Sea. We also postulate that rates of formation of this Fe-P phase are linked to recent changes in methane dynamics and an upward shift of the zone of anaerobic methane oxidation (AOM) with sulfate. Our work indicates that there is limited or no formation of authigenic Ca-P and that permanent burial of P mainly takes place in the form of Fe-bound P and, to a lesser extent, organic P. Extrapolation of reactive P burial fluxes from 6 sites to the basin scale suggests that ∼13,000 tons of reactive P yr^−1^ are sequestered in Bothnian Sea sediments. Our results suggest that the deep basins of the Bothnian Sea are responsible for a major proportion of this P burial.

## Supporting Information

File S1 (DOC)
