# Supplementary material for: Coupled Dynamics of Iron and Phosphorus in Sediments of an Oligotrophic Coastal Basin and the Impact of Anaerobic Oxidation of Methane
Source: PLoS One. 2013 Apr 23;8(4):e62386. doi: 10.1371/journal.pone.0062386 (PMC3633846; doi:10.1371/journal.pone.0062386)
Supplement: File S1 — (DOC) [file pone.0062386.s001.doc]

**SUPPORTING INFORMATION PLOSONE**

**TITLE OF PAPER:** Coupled dynamics of iron and phosphorus in sediments of an oligotrophic coastal basin and the impact of anaerobic oxidation of methane

**AUTHORS:** Caroline P. Slomp*, Haydon P. Mort, Tom Jilbert, Daniel C. Reed, Bo G. Gustafsson, Mariette Wolthers

*Corresponding author: [c.p.slomp@uu.nl](mailto:c.p.slomp@uu.nl).

Department of Earth Sciences – Geochemistry, Faculty of Geosciences, Utrecht University, The Netherlands

**FIGURE A**

**TABLES S1-S7**

**Figure A.** Porewater profiles of sulfate for site US5B in June 2009 and October 2008 as determined by ion chromatography (IC) and ICP-OES.

**Table S1. Porewater profiles (2008 and/or 2009). N.a.: not available due to limited sample volume. SO4: ICP-OES data.**

|  | umol/l | umol/l | mM | umol/l | umol/l |
| --- | --- | --- | --- | --- | --- |
| depth (cm) | Mn | Fe | SO4 | NH4 | PO4 |
| US2 – 2009 |  |  |  |  |  |
| 0 | 1.3 | 3.9 | 5.5 | 4.0 | 2.5 |
| 0.25 | 0.4 | 5.0 | 5.4 | 4.2 | 4.3 |
| 0.75 | 0.4 | 4.5 | 5.4 | 5.3 | 4.8 |
| 1.25 | 0.4 | 3.4 | 5.4 | 5.6 | 5.1 |
| 1.75 | 0.4 | 3.0 | 5.5 | 6.1 | 5.1 |
| 2.5 | 3.6 | 5.4 | 5.5 | 5.1 | 5.4 |
| 3.5 | 52.0 | 2.9 | 5.5 | 7.0 | 5.8 |
| 4.5 | 187.6 | 3.9 | 5.4 | 20.9 | 5.2 |
| 5.5 | 234.5 | 35.2 | 5.4 | 25.0 | 8.0 |
| 6.5 | 181.8 | 116.3 | 5.3 | 44.9 | 30.6 |
| 7.5 | 155.3 | 153.8 | 5.2 | 27.2 | 53.6 |
| 8.5 | 153.5 | 136.3 | 5.2 | 36.9 | 66.0 |
| 9.5 | 160.7 | 118.8 | 5.1 | 32.0 | 79.0 |
| 11 | 200.0 | 91.1 | 5.0 | 35.0 | 81.1 |
| 13 | 240.9 | 40.7 | 4.7 | 50.6 | 98.1 |
| 15 | 324.5 | 21.6 | 3.9 | 115.5 | 140.4 |
| 17 | 404.9 | 32.0 | 3.1 | 164.7 | 168.0 |
| 19 | 471.1 | 59.1 | 1.7 | 186.5 | 201.7 |
| 21 | 541.1 | 99.3 | 1.0 | 317.9 | 209.3 |
| 24 | 607.3 | 187.5 | 0.4 | 449.8 | 215.2 |
| 28 | n.a. | n.a. | 0.0 | 620.7 | 192.3 |
| US5B - 2009 |  |  |  |  |  |
| 0 | 3.3 | 16.6 | 5.5 | 7.9 | 2.3 |
| 0.25 | 37.6 | 10.0 | 5.3 | 48.2 | 5.0 |
| 0.75 | 171.3 | 13.8 | 5.3 | 13.3 | 5.9 |
| 1.25 | 306.7 | 7.9 | 5.2 | 25.3 | 7.1 |
| 1.75 | 518.0 | 11.6 | 5.3 | 59.0 | 9.9 |
| 2.5 | 579.1 | 11.6 | 5.0 | 92.6 | 13.6 |
| 3.5 | 473.3 | 174.3 | 4.7 | 135.4 | 85.0 |
| 4.5 | 408.9 | 170.0 | 4.4 | 153.0 | 76.8 |
| 5.5 | 354.0 | 192.9 | 4.0 | 152.1 | 106.8 |
| 6.5 | 303.8 | 167.9 | 3.5 | 199.2 | 118.2 |
| 7.5 | 243.1 | 48.4 | 3.0 | 222.5 | 30.0 |
| 8.5 | 266.0 | 60.5 | 2.6 | 359.9 | 107.5 |
| 9.5 | 253.8 | 65.2 | 2.0 | 341.0 | 141.1 |
| 11 | 249.1 | 28.8 | 0.8 | 418.1 | 225.8 |
| 13 | 265.5 | 15.9 | 0.1 | 657.7 | 262.3 |
| 15 | 273.1 | 44.3 | 0.1 | 752.6 | 169.6 |
| 17 | 316.5 | 170.0 | 0.1 | 804.3 | 122.5 |
| 19 | 331.6 | 320.7 | 0.1 | 895.8 | 57.7 |
| 21 | 352.7 | 472.1 | 0.1 | 951.0 | 50.1 |
| 23 | 386.4 | 555.5 | 0.1 | 1001.7 | 36.5 |
| 25 | 431.3 | 680.5 | 0.1 | 1037.5 | 37.4 |
| 28 | 491.5 | 887.7 | 0.1 | 1058.9 | 41.5 |
|  |  |  |  |  |  |
|  | umol/l | umol/l | mM | umol/l | umol/l |
| depth (cm) | Mn | Fe | SO4 | NH4 | DIP |
| US5B - 2008 |  |  |  |  |  |
| 0 | 0.9 | 0.0 | 5.5 | 0.5 | 1.5 |
| 0.5 | 87.9 | 2.7 | 5.2 | 26.8 | 5.0 |
| 0.75 | 274.1 | 2.1 | 5.0 | 59.1 | 7.7 |
| 1.25 | 504.9 | 2.5 | 4.5 | 106.6 | 15.4 |
| 1.75 | 564.3 | 10.6 | 4.1 | 146.4 | 28.4 |
| 2.5 | 491.1 | 98.5 | 3.4 | 242.7 | 129.2 |
| 3.5 | 448.0 | 58.6 | 2.0 | 353.3 | 235.2 |
| 4.5 | 434.9 | 15.8 | 1.3 | 403.1 | 306.8 |
| 5.5 | 374.4 | 8.4 | 0.9 | 464.6 | 254.2 |
| 6.5 | 419.4 | 4.3 | 0.4 | 526.4 | 292.9 |
| 7.5 | 433.4 | 5.2 | 0.1 | 618.0 | 308.7 |
| 8.5 | 434.3 | 29.9 | 0.1 | 676.8 | 290.1 |
| 9.5 | 431.4 | 198.0 | 0.5 | 777.0 | 217.5 |
| 13 | 468.2 | 580.7 | 0.1 | n.a. | 134.8 |
| 15 | 539.3 | 980.9 | 0.1 | n.a. | 132.5 |
| 17 | 559.2 | 1002.2 | 0.1 | n.a. | 71.5 |
| 19 | 571.4 | 975.2 | 0.1 | n.a. | 43.8 |
| 21 | 617.2 | 1171.8 | 0.1 | n.a. | 64.8 |
| 25 | 649.6 | 1114.7 | 0.1 | 1451.4 | 42.0 |
| 27 | 708.4 | 955.3 | 0.1 | 1527.4 | 36.8 |
|  |  |  |  |  |  |
| SR5 - 2009 |  |  |  |  |  |
| 0 | 0.2 | 1.1 | 5.7 | 4.1 | 1.7 |
| 0.25 | 0.2 | 1.6 | 5.6 | 4.6 | 2.9 |
| 0.75 | 0.2 | 1.6 | 5.7 | 5.4 | 2.8 |
| 1.25 | 0.0 | 2.1 | 5.6 | 5.3 | 3.0 |
| 1.75 | 0.4 | 2.1 | 5.7 | 4.6 | 3.1 |
| 2.5 | 0.2 | 3.0 | 5.6 | 5.1 | 3.9 |
| 3.5 | 17.5 | 2.1 | 5.6 | 7.0 | 4.0 |
| 4.5 | 70.9 | 2.5 | 5.7 | 30.8 | 4.8 |
| 5.5 | 70.4 | 80.9 | 5.6 | 38.2 | 9.0 |
| 6.5 | 60.5 | 135.4 | 5.6 | 46.3 | 16.0 |
| 7.5 | 50.2 | 178.0 | 5.6 | 52.1 | 24.2 |
| 8.5 | 45.3 | 187.7 | 5.4 | 73.9 | 36.9 |
| 9.5 | 47.3 | 169.3 | 5.3 | 75.2 | 30.3 |
| 11 | 42.4 | 181.1 | 5.2 | 86.4 | 50.0 |
| 13 | 46.7 | 136.6 | 4.8 | 133.2 | 50.6 |
| 15 | 43.5 | 100.2 | 4.5 | 176.1 | 60.7 |
| 17 | 49.1 | 116.8 | 4.0 | 212.2 | 121.8 |
| 19 | 53.8 | 93.2 | 3.6 | 228.9 | 140.6 |
| 21 | 59.8 | 87.3 | 3.3 | 260.2 | 167.0 |
| 23 | 62.9 | 92.1 | 3.0 | 293.2 | 199.5 |
| 25 | 66.0 | 92.7 | 2.7 | 303.0 | 224.5 |
| 27 | 70.4 | 95.7 | 2.3 | 335.0 | 274.9 |
| 30 | 71.8 | 88.8 | 1.9 | 370.9 | 302.8 |
|  |  |  |  |  |  |
|  |  |  |  |  |  |
|  | umol/l | umol/l | mM | umol/l | umol/l |
| depth (cm) | Mn | Fe | SO4 | NH4 | DIP |
| SR5- 2008 |  |  |  |  |  |
| 0 | 0.0 | 3.4 | 5.9 | 2.0 | 1.5 |
| 0.25 | 0.0 | 2.3 | 6.0 | 2.0 | 3.7 |
| 0.75 | 0.0 | 2.7 | 5.9 | 5.9 | 4.6 |
| 1.25 | 0.0 | 1.1 | 5.9 | 3.0 | 4.0 |
| 2.5 | 0.0 | 1.1 | 5.9 | 1.0 | 4.4 |
| 3.5 | 1.5 | 0.0 | 5.7 | 10.4 | 5.0 |
| 4.5 | 27.3 | 1.1 | 5.8 | 11.8 | 3.9 |
| 5.5 | 74.1 | 1.8 | 5.8 | 19.8 | 1.8 |
| 6.5 | 62.8 | 102.1 | 5.8 | 36.8 | 10.0 |
| 7.5 | 69.4 | 104.2 | 5.8 | 98.3 | 15.9 |
| 8.5 | 56.1 | 139.9 | 5.7 | 69.3 | 22.7 |
| 9.5 | 41.9 | 139.3 | 5.6 | 74.9 | 32.6 |
| 10.5 | 40.2 | 128.9 | 5.5 | 89.4 | 40.4 |
| 12 | 39.5 | 95.3 | 5.3 | 100.4 | 37.1 |
| 14 | 40.8 | 92.2 | 4.9 | n.a. | 64.2 |
| 16 | 46.4 | 98.3 | 4.7 | 149.6 | 99.2 |
| 18 | 49.3 | 76.3 | 4.3 | 164.2 | 105.7 |
| 20 | 53.9 | 87.6 | 4.0 | n.a. | 148.6 |
| 22 | n.a. | n.a. | n.a. | 112.9 | 130.3 |
| 24 | 63.5 | 94.4 | 3.3 | 223.0 | 210.4 |
|  |  |  |  |  |  |
| F26 - 2009 |  |  |  |  |  |
| 0 | 0.2 | 0.0 | 5.5 | 9.3 | 1.9 |
| 1 | 0.0 | 0.0 | 5.4 | 16.9 | 2.0 |
| 2 | 0.0 | 0.0 | 5.4 | 11.7 | 2.1 |
| 3 | 0.2 | 0.0 | 5.3 | 10.0 | 2.2 |
| 4 | 31.3 | 0.0 | 5.3 | 14.7 | 1.9 |
| 5 | 95.6 | 183.8 | 5.2 | 32.9 | 12.5 |
| 7 | 74.5 | 400.5 | 5.3 | 37.7 | 49.5 |
| 9 | 65.5 | 408.4 | 5.2 | 51.6 | 52.9 |
| 11 | 60.0 | 304.3 | 5.1 | 58.6 | 39.2 |
| 13 | 86.4 | 375.4 | 4.7 | 92.6 | 68.9 |
| 15 | 112.4 | 400.0 | 4.3 | 131.7 | 99.6 |
| 18 | 136.9 | 413.6 | 3.8 | 179.6 | 128.3 |
| 21 | 143.8 | 396.1 | 3.4 | 210.0 | 134.7 |
| 24 | 156.5 | 425.7 | 2.9 | 242.0 | 164.7 |
| 27 | 160.5 | 421.4 | 2.6 | 284.1 | 176.3 |
| 30 | 165.5 | 402.1 | 2.1 | 324.0 | 188.8 |
| 36 | 170.0 | 330.7 | 1.2 | 371.6 | 214.3 |
| F26 - 2008 |  |  |  |  |  |
| 0 | 0.0 | 0.9 | 5.6 | 1.5 | 1.6 |
| 1 | 0.5 | 0.0 | 5.6 | 12.9 | 1.7 |
| 2 | 0.4 | 2.3 | 5.5 | 11.9 | 2.1 |
| 3 | 21.1 | 0.0 | 5.6 | 14.3 | 1.8 |
| 4 | 34.9 | 43.0 | 5.5 | 12.9 | 2.5 |
| 5 | 82.1 | 365.3 | 5.7 | 33.1 | 28.2 |
| 6 | 77.7 | 420.6 | 5.6 | 42.5 | 30.0 |
|  | umol/l | umol/l | mM | umol/l | umol/l |
| depth (cm) | Mn | Fe | SO4 | NH4 | DIP |
| F26 – 2008  continued |  |  |  |  |  |
| 8 | 63.3 | 281.1 | 5.4 | n.a. | 27.8 |
| 10 | 86.5 | 362.4 | 5.5 | 80.1 | 37.2 |
| 12 | 98.8 | 255.2 | 4.5 | n.a. | 34.7 |
| 14 | 104.8 | 305.1 | 4.6 | 124.1 | 76.7 |
| 17 | 130.1 | 354.7 | 4.1 | 167.6 | 101.2 |
| 19 | 133.8 | 352.8 | 3.9 | 177.5 | 107.9 |
| 30 | 192.6 | 446.2 | 2.1 | 296.6 | 178.8 |
| 35 | 181.7 | 298.0 | 1.1 | n.a. | 169.8 |
|  |  |  |  |  |  |
| SR7 - 2008 |  |  |  |  |  |
| 0 | 0.0 | 0.9 | 5.1 | 2.0 | 1.0 |
| 0.5 | 0.0 | 0.0 | 5.2 | 3.5 | 2.1 |
| 1 | 2.4 | 0.0 | 5.3 | 19.8 | 2.8 |
| 4 | 64.1 | 1.4 | 5.6 | 32.6 | 1.1 |
| 7 | 57.3 | 6.6 | 5.4 | 26.2 | 2.3 |
| 8 | 60.8 | 10.9 | 5.7 | 25.7 | 3.3 |
| 11 | 56.8 | 3.9 | 5.5 | n.a. | 3.5 |
| 14 | 47.1 | 21.3 | 5.6 | 23.7 | 7.8 |
| 17 | 39.3 | 19.5 | 5.5 | 32.1 | 7.1 |
| 20 | 28.9 | 29.4 | 5.5 | 26.2 | 10.2 |
| 26 | 18.0 | 29.7 | 5.4 | 41.0 | 11.0 |
| 30 | 14.7 | 41.5 | 5.5 | 22.2 | 16.2 |
| SR1a - 2008 |  |  |  |  |  |
| 0 | 0.4 | 1.1 | 4.7 | 0.0 | 3.0 |
| 1 | 4.2 | 2.1 | 4.8 | 11.4 | 1.6 |
| 2 | 10.4 | 2.9 | 4.7 | 25.2 | 1.2 |
| 3 | 12.6 | 12.2 | 4.6 | 14.3 | 1.1 |
| 4 | 9.8 | 8.6 | 4.7 | 18.3 | 0.9 |
| 6 | 13.5 | 17.5 | 4.7 | 21.3 | 1.6 |
| 11 | 29.1 | 46.0 | 4.6 | 66.2 | n.a. |
| 13 | 14.6 | 19.7 | 4.8 | 44.0 | 6.2 |
| 18 | 29.7 | 16.7 | 4.8 | 23.2 | 2.4 |

**Table S2. Pore water methane profiles for US5B and US2 in 2009.**

| **Site** | Sample interval | Mid-depth (cm) | CH4 (umol/l) |
| --- | --- | --- | --- |
| **US2** | 3-5cm | 4 | 0 |
|  | 8-10cm | 9 | 0 |
|  | 13-15cm | 14 | 0 |
|  | 18-20cm | 19 | 324 |
|  | 23-25cm | 24 | 950 |
|  | 28-30cm | 29 | 1618 |
|  | 33-35cm | 34 | 1921 |
|  | 38-40cm | 39 | 2247 |
|  |  |  |  |
| **US5B** | 3-5cm | 4 | 0 |
|  | 8-10cm | 9 | 355 |
|  | 13-15cm | 14 | 977 |
|  | 18-20cm | 19 | 1907 |
|  | 23-25cm | 24 | 2611 |
|  | 28-30cm | 29 | 2838 |
|  | 33-35cm | 34 | 3078 |
|  | 38-40cm | 39 | 2985 |

**Table S3. Sediment depth profiles of CDB Fe and total Fe, Mn and S. Units in μmol/g.**

| Site | Depth (cm) | CDB-Fe (umol/g) | Fe-tot (umol/g) | Mn-tot (umol/g) | S tot (umol/g) |
| --- | --- | --- | --- | --- | --- |
| US 2 | 0.25 | 350.0 | 1116 | 172 | 24 |
|  | 0.75 | 348.8 | 1101 | 175 | 22 |
|  | 1.25 | 324.2 | 1099 | 168 | 23 |
|  | 1.75 | 348.8 | 1124 | 172 | 24 |
|  | 2.5 | 379.5 | 1139 | 180 | 24 |
|  | 3.5 | 388.5 | 1133 | 201 | 25 |
|  | 4.5 | 441.4 | 1147 | 125 | 23 |
|  | 5.5 | 453.2 | 1193 | 54 | 27 |
|  | 6.5 | 347.9 | 1052 | 39 | 39 |
|  | 7.5 | 264.0 | 1047 | 35 | 74 |
|  | 8.5 | 245.3 | 1031 | 32 | 126 |
|  | 9.5 | 267.4 | 1077 | 42 | 130 |
|  | 11 | 266.8 | 1059 | 51 | 150 |
|  | 13 | 268.9 | 1073 | 47 | 237 |
|  | 15 | 269.2 | 1097 | 45 | 312 |
|  | 17 | 265.3 | 1068 | 52 | 234 |
|  | 19 | 266.4 | 1053 | 48 | 200 |
|  | 21 | 258.6 | 1086 | 50 | 152 |
|  | 24 | 254.7 | 1062 | 49 | 106 |
|  | 28 | 288.9 | 1074 | 56 | 79 |
|  | 32 | 344.1 | 1087 | 56 | 105 |
|  |  |  |  |  |  |
| US5B | 0.50 | 239.0 | 1140 | 137 | 34 |
|  | 0.75 | 235.4 | 1123 | 108 | 37 |
|  | 1.25 | 290.8 | 1144 | 86 | 36 |
|  | 1.75 | 212.8 | 1175 | 69 | 31 |
|  | 2.50 | 275.3 | 1144 | 65 | 32 |
|  | 3.5 | 140.8 | 1079 | 48 | 82 |
|  | 4.5 | 251.7 | 1193 | 39 | 373 |
|  | 5.5 | 239.6 | 910 | 24 | 419 |
|  | 6.5 | 300.0 | 1215 | 35 | 471 |
|  | 7.5 | 298.3 | 1217 | 35 | 436 |
|  | 8.5 | 393.7 | 1302 | 34 | 519 |
|  | 9.5 | 157.0 | 1105 | 34 | 121 |
|  | 11.0 | 106.6 | 933 | 27 | 56 |
|  | 13.0 | 104.2 | 890 | 22 | 39 |
|  | 15.0 | 98.5 | 751 | 18 | 28 |
|  | 17.0 | 136.2 | 1091 | 26 | 39 |
|  | 19.0 | 107.9 | 915 | 23 | 38 |
|  | 21.0 | 155.2 | 1169 | 27 | 49 |
|  | 23.0 | 88.8 | 933 | 23 | 54 |
|  | 25.0 | 200.8 | 1171 | 27 | 116 |
|  | 27.0 | 301.5 | 1238 | 29 | 172 |
|  | 32.0 | 139.2 | 1037 | 27 | 144 |
|  |  |  |  |  |  |
|  |  |  |  |  |  |
| Site | Depth (cm) | CDB-Fe (umol/g) | Fe-tot (umol/g) | Mn-tot (umol/g) | S tot (umol/g) |
| SR5 | 0.25 | 315.4 | 1208 | 55 | 44 |
|  | 0.75 | 333.6 | 1223 | 56 | 42 |
|  | 1.25 | 368.9 | 1279 | 60 | 39 |
|  | 1.75 | 363.8 | 1246 | 62 | 40 |
|  | 2.5 | 266.5 | 1225 | 62 | 40 |
|  | 3.5 | 289.8 | 1019 | 54 | 33 |
|  | 4.5 | 273.7 | 1065 | 42 | 31 |
|  | 5.5 | 173.1 | 1142 | 23 | 35 |
|  | 6.5 | 88.2 | 1026 | 11 | 57 |
|  | 7.5 | 85.1 | 944 | 10 | 55 |
|  | 8.5 | 98.0 | 1039 | 12 | 72 |
|  | 9.5 | 116.8 | 1030 | 14 | 74 |
|  | 10.5 | 113.3 | 1069 | 17 | 83 |
|  | 12.0 | 127.4 | 1058 | 15 | 95 |
|  | 14.0 | 97.9 | 997 | 11 | 96 |
|  | 16.0 | 76.6 | 980 | 11 | 85 |
|  | 18.0 | 63.8 | 1023 | 11 | 77 |
|  | 20.0 | 60.2 | 1028 | 11 | 79 |
|  | 22.0 | 66.9 | 995 | 11 | 60 |
|  | 24.0 | 48.0 | 1009 | 11 | 43 |
|  |  |  |  |  |  |
| F 26 | 0.25 | 316.7 | 1297 | 81 | 51.2 |
|  | 0.75 | 346.5 | 1355 | 89 | 44.1 |
|  | 1.25 | 348.2 | 1304 | 92 | 44.8 |
|  | 1.75 | 312.8 | 1280 | 91 | 45.8 |
|  | 2.5 | 353.1 | 1362 | 101 | 42.9 |
|  | 3.5 | 329.8 | 1302 | 97 | 39.7 |
|  | 4.5 | 313.4 | 1396 | 82 | 39.8 |
|  | 5.5 | 423.3 | 1241 | 26 | 40.7 |
|  | 6.5 | 208.8 | 1219 | 22 | 42.6 |
|  | 7.5 | 108.1 | 1074 | 15 | 51.8 |
|  | 8.5 | 142.6 | 1091 | 19 | 50.2 |
|  | 9.5 | 100.9 | 1009 | 14 | 53.2 |
|  | 11 | 113.1 | 952 | 16 | 53.6 |
|  | 13 | 138.4 | 1128 | 17 | 79.2 |
|  | 15 | 124.3 | 1136 | 18 | 77.7 |
|  | 17 | 116.5 | 1151 | 17 | 77.7 |
|  | 19 | 107.3 | 1126 | 15 | 75.1 |
|  | 21 | 113.9 | 1115 | 16 | 78.3 |
|  | 23 | 105.1 | 1062 | 14 | 72.9 |
|  | 25 | 91.5 | 1083 | 15 | 66.5 |
|  | 27 | 96.8 | 1136 | 15 | 68.6 |
|  | 29 | 100.4 | 1112 | 16 | 65.9 |
|  | 31 | 90.5 | 1136 | 15 | 69.6 |
|  | 33 | 139.1 | 1203 | 24 | 82.9 |
|  | 35 | 77.4 | 1108 | 14 | 72.2 |
|  |  |  |  |  |  |
|  |  |  |  |  |  |
|  | Depth (cm) | CDB-Fe (umol/g) | Fe-tot (umol/g) | Mn-tot (umol/g) | S tot (umol/g) |
| SR 7 | 0.50 | 100.9 | 595 | 193 | 23 |
|  | 1.25 | 96.7 | 601 | 208 | 21 |
|  | 1.75 | 78.8 | 626 | 222 | 22 |
|  | 2.50 | 101.0 | 544 | 214 | 18 |
|  | 3.5 | 79.3 | 539 | 185 | 17 |
|  | 4.5 | 68.6 | 440 | 99 | 12 |
|  | 5.5 | 47.6 | 455 | 20 | 9 |
|  | 6.5 | 61.5 | 765 | 78 | 17 |
|  | 7.5 | 163.4 | 1022 | 23 | 53 |
|  | 8.5 | 54.4 | 1115 | 16 | 668 |
|  | 9.5 | 32.5 | 1149 | 16 | 701 |
|  | 11 | 12.0 | 1245 | 16 | 845 |
|  | 13 | 6.8 | 1022 | 14 | 300 |
|  | 15 | 8.4 | 1195 | 15 | 627 |
|  | 17 | 11.0 | 927 | 13 | 355 |
|  | 19 | 10.7 | 1488 | 17 | 1369 |
|  | 21 | 10.6 | 1149 | 15 | 567 |
|  | 23 | 14.9 | 966 | 11 | 34 |
|  | 25 | 20.7 | 986 | 11 | 29 |
|  | 27 | 18.8 | 974 | 9 | 282 |
|  | 29 | 21.3 | 1258 | 10 | 555 |
|  | 31 | 29.0 | 1230 | 10 | 450 |
|  |  |  |  |  |  |
| SR1 a | 0.5 | 169.2 | 795 | 26.1 | 54 |
|  | 1.25 | 156.5 | 832 | 27.9 | 55 |
|  | 1.75 | 176.8 | 757 | 26.7 | 60 |
|  | 2.5 | 161.6 | 691 | 24.0 | 54 |
|  | 3.5 | 77.3 | 739 | 16.7 | 150 |
|  | 4.5 | 19.3 | 1015 | 12.0 | 387 |
|  | 5.5 | 16.4 | 1046 | 12.3 | 364 |
|  | 6.5 | 8.2 | 1083 | 12.6 | 391 |
|  | 7.5 | 8.5 | 1067 | 11.9 | 464 |
|  | 8.5 | 7.8 | 1014 | 11.8 | 355 |
|  | 9.5 | 7.8 | 1009 | 11.8 | 371 |
|  | 11 | 10.3 | 1066 | 12.5 | 405 |
|  | 13 | 6.1 | 1108 | 13.9 | 465 |
|  | 15 | 6.0 | 1044 | 12.6 | 466 |
|  | 17 | 5.3 | 1097 | 12.9 | 523 |
|  | 19 | 5.7 | 997 | 11.5 | 351 |
|  | 21 | 5.0 | 1021 | 11.4 | 425 |
|  | 23 | 4.5 | 1001 | 11.3 | 408 |

**Table S4. Depth profiles of sediment P forms (in μmol/g) and organic C (wt%)**

|  |  | Ex-P | Fe-P | Auth-P | Detr-P | Org-P | Tot-P | Corg |
| --- | --- | --- | --- | --- | --- | --- | --- | --- |
|  | Depth (cm) | umol/g | umol/g | umol/g | umol/g | umol/g | umol/g | % |
| US2 | 0.25 | 2.68 | 46.85 | 12.20 | 2.75 | 7.44 | 71.92 | 3.07 |
|  | 0.75 | 3.26 | 46.59 | 12.29 | 2.38 | 7.34 | 71.85 | 3.15 |
|  | 1.25 | 3.49 | 42.71 | 12.00 | 2.35 | 7.41 | 67.96 | 3.07 |
|  | 1.75 | 3.51 | 45.60 | 11.83 | 2.42 | 7.15 | 70.50 | 3.06 |
|  | 2.5 | 3.69 | 49.95 | 12.34 | 2.36 | 6.91 | 75.26 | 3.09 |
|  | 3.5 | 2.84 | 52.04 | 11.36 | 3.21 | 7.38 | 76.84 | 3.23 |
|  | 4.5 | 3.54 | 63.58 | 12.56 | 2.85 | 7.80 | 90.33 | 3.29 |
|  | 5.5 | 3.69 | 70.57 | 12.76 | 3.07 | 7.70 | 97.78 | 3.30 |
|  | 6.5 | 3.26 | 36.64 | 11.30 | 2.45 | 8.78 | 62.43 | 3.33 |
|  | 7.5 | 1.80 | 17.09 | 11.83 | 2.50 | 6.94 | 40.15 | 3.06 |
|  | 8.5 | 0.99 | 13.71 | 11.98 | 2.52 | 7.28 | 36.48 | 2.88 |
|  | 9.5 | 1.04 | 19.52 | 12.28 | 2.76 | 7.20 | 42.80 | 2.82 |
|  | 11 | 0.82 | 24.05 | 12.31 | 2.48 | 6.67 | 46.32 | 2.51 |
|  | 13 | 0.44 | 15.38 | 12.31 | 2.58 | 6.80 | 37.51 | 2.44 |
|  | 15 | 0.31 | 14.61 | 12.62 | 2.32 | 6.79 | 36.65 | 2.47 |
|  | 17 | 0.49 | 21.94 | 13.25 | 2.54 | 6.63 | 44.85 | 2.47 |
|  | 19 | 0.47 | 19.09 | 11.47 | 3.50 | 6.95 | 41.48 | 2.60 |
|  | 21 | 0.77 | 23.44 | 12.20 | 2.92 | 6.96 | 46.30 | 2.55 |
|  | 24 | 0.91 | 26.42 | 11.70 | 2.96 | 6.84 | 48.82 | 2.51 |
|  | 28 | 1.05 | 38.91 | 12.69 | 3.14 | 7.29 | 63.07 | 2.60 |
|  | 32 | 0.96 | 39.36 | 12.28 | 2.99 | 7.04 | 62.63 | 2.64 |
|  |  |  |  |  |  |  |  |  |
| US5 b | 0.5 | 0.97 | 31.74 | 4.48 | 1.70 | 7.48 | 46.37 | 2.66 |
|  | 0.75 | 1.11 | 32.07 | 4.21 | 1.68 | 7.57 | 46.64 | 2.73 |
|  | 1.25 | 1.40 | 38.84 | 4.46 | 1.57 | 7.92 | 54.18 | 2.92 |
|  | 1.75 | 2.14 | 36.40 | 4.46 | 1.56 | 8.11 | 52.67 | 2.75 |
|  | 2.5 | 2.17 | 50.53 | 4.34 | 2.02 | 7.33 | 66.39 | 2.87 |
|  | 3.5 | 1.67 | 20.16 | 4.56 | 1.50 | 7.37 | 35.26 | 2.67 |
|  | 4.5 | 0.27 | 11.76 | 4.66 | 1.35 | 7.11 | 25.15 | 2.60 |
|  | 5.5 | 0.08 | 8.12 | 2.90 | 0.63 | 6.44 | 18.17 | 2.09 |
|  | 6.5 | 0.15 | 12.96 | 4.39 | 1.33 | 6.02 | 24.84 | 2.27 |
|  | 7.5 | 0.20 | 10.47 | 4.35 | 1.26 | 6.28 | 22.56 | 2.23 |
|  | 8.5 | 0.10 | 22.26 | 4.37 | 1.41 | 5.98 | 34.12 | 2.25 |
|  | 9.5 | 0.30 | 26.41 | 4.46 | 1.79 | 6.26 | 39.23 | 2.40 |
|  | 11 | 0.39 | 20.27 | 3.74 | 1.27 | 6.76 | 32.43 | 2.33 |
|  | 13 | 0.38 | 19.22 | 3.19 | 1.91 | 6.28 | 30.97 | 2.28 |
|  | 15 | 0.47 | 17.22 | 2.70 | 0.98 | 5.56 | 26.92 | 2.22 |
|  | 17 | 0.32 | 22.68 | 3.82 | 1.95 | 5.62 | 34.40 | 2.31 |
|  | 19 | 0.47 | 21.02 | 3.05 | 0.89 | 6.37 | 31.81 | 2.17 |
|  | 21 | 0.30 | 28.43 | 3.40 | 2.25 | 6.64 | 41.03 | 2.42 |
|  | 23 | 0.54 | 13.43 | 2.71 | 1.24 | 5.85 | 23.78 | 2.01 |
|  | 25 | 0.25 | 25.21 | 4.26 | 1.53 | 6.63 | 37.89 | 2.28 |
|  | 27 | 0.26 | 32.44 | 3.90 | 1.87 | 6.13 | 44.60 | 2.25 |
|  | 32 | 0.52 | 16.88 | 3.00 | 0.88 | 6.11 | 27.38 | 2.04 |
|  |  |  |  |  |  |  |  |  |
|  |  | Ex-P | Fe-P | Auth-P | Detr-P | Org-P | Tot-P | Corg |
|  | Depth (cm) | umol/g | umol/g | umol/g | umol/g | umol/g | umol/g | % |
| SR 5 | 0.25 | 1.32 | 44.30 | 3.90 | 2.46 | 9.13 | 61.11 | 2.66 |
|  | 0.75 | 0.76 | 48.24 | 3.60 | 3.18 | 8.23 | 64.01 | 2.69 |
|  | 1.25 | 0.76 | 54.29 | 3.46 | 3.32 | 8.16 | 69.99 | 2.76 |
|  | 1.75 | 1.02 | 53.33 | 4.22 | 2.11 | 7.85 | 68.53 | 2.80 |
|  | 2.5 | 0.94 | 38.04 | 3.30 | 1.55 | 7.99 | 51.81 | 2.81 |
|  | 3.5 | 0.85 | 42.41 | 3.41 | 1.83 | 8.57 | 57.08 | 2.88 |
|  | 4.5 | 1.00 | 40.63 | 3.42 | 1.36 | 9.38 | 55.78 | 2.79 |
|  | 5.5 | 0.64 | 24.38 | 3.59 | 2.20 | 8.20 | 39.01 | 2.67 |
|  | 6.5 | 0.15 | 6.91 | 3.61 | 3.41 | 7.09 | 21.17 | 2.51 |
|  | 7.5 | 0.18 | 6.31 | 3.48 | 2.90 | 7.55 | 20.42 | 2.41 |
|  | 8.5 | 0.16 | 6.55 | 3.83 | 2.73 | 7.27 | 20.54 | 2.49 |
|  | 9.5 | 0.30 | 20.72 | 3.91 | 2.65 | 7.76 | 35.34 | 2.49 |
|  | 10.5 | 0.16 | 25.76 | 4.71 | 1.96 | 8.73 | 41.32 | 2.44 |
|  | 12 | 0.18 | 21.87 | 4.67 | 2.12 | 7.74 | 36.60 | 2.46 |
|  | 14 | 0.23 | 8.12 | 4.35 | 1.82 | 7.88 | 22.40 | 2.42 |
|  | 16 | 0.22 | 7.20 | 4.75 | 1.95 | 7.67 | 21.79 | 2.31 |
|  | 18 | 0.33 | 5.80 | 4.47 | 2.03 | 8.05 | 20.68 | 2.25 |
|  | 20 | 0.35 | 4.88 | 4.93 | 1.51 | 7.74 | 19.40 | 1.46 |
|  | 22 | 0.33 | 4.75 | 4.16 | 2.48 | 6.56 | 18.28 | 2.07 |
|  | 24 | 0.28 | 4.67 | 4.83 | 2.14 | 6.25 | 18.16 | 1.77 |
|  |  |  |  |  |  |  |  |  |
| F 26 | 0.25 | 0.95 | 37.42 | 5.10 | 1.02 | 3.49 | 47.98 | 2.29 |
|  | 0.75 | 1.02 | 40.12 | 5.20 | 1.19 | 3.73 | 51.25 | n.a. |
|  | 1.25 | 1.29 | 40.62 | 5.12 | 1.16 | 4.02 | 52.21 | 2.90 |
|  | 1.75 | 1.30 | 35.24 | 4.86 | 0.96 | 3.47 | 45.83 | 3.36 |
|  | 2.5 | 1.14 | 41.11 | 4.95 | 1.28 | 3.61 | 52.10 | 3.10 |
|  | 3.5 | 1.09 | 38.77 | 4.56 | 0.98 | 3.24 | 48.64 | 3.00 |
|  | 4.5 | 1.14 | 36.18 | 4.89 | 1.25 | 3.61 | 47.07 | 3.02 |
|  | 5.5 | 0.92 | 49.21 | 5.20 | 1.53 | 4.00 | 60.85 | 2.80 |
|  | 6.5 | 0.48 | 34.77 | 4.75 | 2.82 | 4.37 | 47.17 | 2.63 |
|  | 7.5 | 0.32 | 11.67 | 4.23 | 2.47 | 5.22 | 23.92 | 2.65 |
|  | 8.5 | 0.66 | 36.31 | 5.56 | 1.88 | 3.83 | 48.23 | 2.65 |
|  | 9.5 | 0.42 | 16.92 | 5.05 | 1.81 | 3.79 | 27.99 | 2.67 |
|  | 11 | 0.47 | 29.38 | 4.73 | 1.54 | 3.13 | 39.25 | 2.70 |
|  | 13 | 0.19 | 25.38 | 5.96 | 2.08 | 4.19 | 37.80 | 2.68 |
|  | 15 | 0.22 | 21.58 | 5.44 | 3.32 | 4.30 | 34.87 | 2.47 |
|  | 17 | 0.15 | 15.62 | 5.89 | 1.87 | 3.74 | 27.28 | 2.43 |
|  | 19 | 0.14 | 8.28 | 5.67 | 1.59 | 3.22 | 18.90 | 2.23 |
|  | 21 | 0.12 | 10.72 | 6.03 | 1.68 | 3.29 | 21.83 | 2.21 |
|  | 23 | 0.26 | 7.18 | 5.04 | 1.50 | 2.95 | 16.93 | 2.12 |
|  | 25 | 0.20 | 10.73 | 5.65 | 1.75 | 3.26 | 21.59 | 1.93 |
|  | 27 | 0.15 | 9.81 | 5.82 | 1.76 | 3.12 | 20.66 | 1.89 |
|  | 29 | 0.25 | 13.19 | 5.71 | 1.78 | 3.13 | 24.06 | 1.85 |
|  | 31 | 0.19 | 6.39 | 5.84 | 1.91 | 3.36 | 17.69 | 1.77 |
|  | 33 | 0.29 | 36.44 | 7.72 | 2.12 | 2.62 | 49.19 | 1.83 |
|  | 35 | 0.15 | 6.01 | 5.42 | 1.81 | 2.67 | 16.07 | 1.74 |
|  |  |  |  |  |  |  |  |  |
|  |  | Ex-P | Fe-P | Auth-P | Detr-P | Org-P | Tot-P | Corg |
|  | Depth (cm) | umol/g | umol/g | umol/g | umol/g | umol/g | umol/g | % |
| SR 7 | 0.5 | 0.16 | 12.23 | 3.39 | 2.58 | 1.87 | 20.23 | 1.30 |
|  | 1.25 | 0.16 | 10.87 | 3.62 | 2.85 | 1.97 | 19.47 | 1.33 |
|  | 1.75 | 0.18 | 7.96 | 3.71 | 2.79 | 1.84 | 16.48 | 1.27 |
|  | 2.5 | 0.15 | 11.56 | 3.15 | 2.90 | 1.72 | 19.48 | 1.02 |
|  | 3.5 | 0.12 | 8.66 | 3.20 | 3.77 | 1.75 | 17.50 | 0.75 |
|  | 4.5 | 0.12 | 7.28 | 3.64 | 4.33 | 1.32 | 16.69 | 0.55 |
|  | 5.5 | 0.04 | 4.79 | 4.16 | 4.51 | 1.02 | 14.52 | 0.36 |
|  | 6.5 | 0.10 | 5.81 | 5.55 | 2.99 | 1.88 | 16.34 | 1.05 |
|  | 7.5 | 0.17 | 20.50 | 5.71 | 2.89 | 2.20 | 31.47 | 1.35 |
|  | 8.5 | 0.03 | 4.49 | 3.34 | 3.21 | 1.86 | 12.94 | 0.91 |
|  | 9.5 | 0.03 | 2.33 | 4.87 | 2.73 | 1.76 | 11.72 | 1.07 |
|  | 11 | 0.01 | 1.22 | 4.71 | 1.70 | 2.81 | 10.46 | 1.40 |
|  | 13 | 0.05 | 1.93 | 4.96 | 1.52 | 3.45 | 11.90 | 1.72 |
|  | 15 | 0.03 | 1.64 | 4.89 | 1.53 | 3.81 | 11.89 | 1.84 |
|  | 17 | 0.03 | 1.76 | 4.15 | 1.39 | 3.52 | 10.85 | 1.98 |
|  | 19 | 0.05 | 2.06 | 4.36 | 1.78 | 3.21 | 11.45 | 1.49 |
|  | 21 | 0.08 | 2.22 | 4.75 | 1.40 | 3.38 | 11.83 | 1.37 |
|  | 23 | 0.04 | 2.40 | 4.68 | 1.50 | 3.16 | 11.79 | 1.33 |
|  | 25 | 0.09 | 3.10 | 2.43 | 3.27 | 2.22 | 11.12 | 0.18 |
|  | 27 | 0.07 | 2.67 | 2.85 | 2.85 | 1.58 | 10.03 | n.a. |
|  | 29 | 0.13 | 2.76 | 2.73 | 3.94 | 1.45 | 11.00 | 0.33 |
|  | 31 | 0.19 | 3.24 | 3.71 | 4.24 | 1.08 | 12.46 | 0.22 |
|  |  |  |  |  |  |  |  |  |
| SR 1a | 0.5 | 0.32 | 13.02 | 4.97 | 6.65 | 4.82 | 29.78 | 1.33 |
|  | 1.25 | 0.33 | 11.55 | 4.85 | 5.88 | 4.81 | 27.41 | 1.33 |
|  | 1.75 | 0.43 | 13.82 | 5.03 | 6.57 | 4.52 | 30.36 | 0.78 |
|  | 2.5 | 0.18 | 11.55 | 3.97 | 8.62 | 3.69 | 28.01 | 0.81 |
|  | 3.5 | 0.20 | 4.88 | 5.20 | 9.32 | 3.34 | 22.94 | 0.74 |
|  | 4.5 | 0.08 | 2.08 | 7.71 | 4.35 | 4.67 | 18.89 | 1.90 |
|  | 5.5 | 0.12 | 2.11 | 7.80 | 3.81 | 4.92 | 18.76 | 2.05 |
|  | 6.5 | 0.10 | 1.70 | 7.99 | 3.49 | 4.54 | 17.82 | 2.06 |
|  | 7.5 | 0.14 | 1.73 | 8.57 | 3.43 | 4.76 | 18.63 | 2.13 |
|  | 8.5 | 0.90 | 2.06 | 8.34 | 3.24 | 5.00 | 19.54 | 2.17 |
|  | 9.5 | 0.15 | 1.87 | 8.74 | 3.23 | 5.61 | 19.60 | n.a. |
|  | 11 | 0.27 | 3.05 | 8.19 | 3.73 | 5.96 | 21.21 | 2.29 |
|  | 13 | 0.08 | 1.76 | 8.56 | 3.25 | 5.75 | 19.42 | 2.25 |
|  | 15 | 0.12 | 1.76 | 8.08 | 3.11 | 5.55 | 18.63 | 2.24 |
|  | 17 | 0.06 | 1.94 | 8.67 | 3.35 | 5.78 | 19.81 | 2.44 |
|  | 19 | 0.04 | 1.81 | 8.39 | 3.18 | 5.41 | 18.84 | 2.43 |
|  | 21 | 0.11 | n.a. | 8.16 | 3.44 | 5.64 | 17.35 | 2.50 |
|  | 23 | 0.11 | n.a. | 8.34 | 3.38 | 5.99 | 17.82 | 2.82 |

**Table S5. Depth profiles of sediment P, Fe and Mn for sites US5B and SR5, extracted with ascorbate and CDB (in μmol/g).**

| depth (cm) | umol/g | umol/g | umol/g |
| --- | --- | --- | --- |
| US5B - Oct 2008 | Ascorbate-Fe | Ascorbate-Mn | Ascorbate-P |
| 0.5 | 176.0 | 73.9 | 29.7 |
| 1.25 | 187.6 | 41.0 | 36.2 |
| 2.5 | 200.1 | 32.9 | 47.3 |
| 3.5 | 96.4 | 19.0 | 23.1 |
| 4.5 | 183.7 | 13.8 | 11.7 |
| 9.5 | 121.0 | 14.9 | 27.8 |
| 21 | 107.3 | 10.1 | 26.3 |
|  |  |  |  |
| SR5 - Oct 2008 |  |  |  |
| 0.75 | 263.2 | 33.7 | 45.3 |
| 1.25 | 267.2 | 34.4 | 45.9 |
| 2.5 | 228.0 | 32.7 | 40.0 |
| 3.5 | 249.71 | 38.71 | 44.46 |
| 6.5 | 39.1 | 1.0 | 8.5 |
| 10.5 | 83.3 | 4.9 | 28.4 |
| 12 | 71.5 | 3.5 | 21.4 |
| 20 | 30.4 | 0.9 | 7.1 |
|  |  |  |  |
|  | umol/g | umol/g | umol/g |
| US5B - Oct 2008 | CDB-Fe | CDB-Mn | CDB-P |
| 0.5 | 239 | 82.0 | 31.7 |
| 0.75 | 235 | 57.8 | 32.1 |
| 1.25 | 291 | 50.7 | 38.8 |
| 1.75 | 213 | 21.4 | 36.4 |
| 2.5 | 275 | 29.1 | 50.5 |
| 3.5 | 141 | 16.5 | 20.2 |
| 4.5 | 252 | 16.2 | 11.8 |
| 5.5 | 240 | 6.7 | 8.1 |
| 6.5 | 300 | 15.4 | 13.0 |
| 7.5 | 298 | 14.5 | 10.5 |
| 8.5 | 394 | 18.4 | 22.3 |
| 9.5 | 157 | 13.0 | 26.4 |
| 11 | 107 | 8.3 | 20.3 |
| 13 | 104 | 7.2 | 19.2 |
| 15 | 98 | 5.5 | 17.2 |
| 17 | 136 | 7.9 | 22.7 |
| 19 | 108 | 5.6 | 21.0 |
| 21 | 155 | 9.5 | 28.4 |
| 23 | 89 | 4.1 | 13.4 |
| 25 | 201 | 10.7 | 25.2 |
| 27 | 301 | 15.2 | 32.4 |
| 32 | 139 | 4.9 | 16.9 |
|  |  |  |  |
|  |  |  |  |
|  | umol/g | umol/g | umol/g |
| SR5 - Oct 2008 | CDB-Fe | CDB-Mn | CDB-P |
| 0.25 | 315 | 36.3 | 44.3 |
| 0.75 | 334 | 38.1 | 48.2 |
| 1.25 | 369 | 44.0 | 54.3 |
| 1.75 | 364 | 45.8 | 53.3 |
| 2.5 | 266 | 34.4 | 38.0 |
| 3.5 | 290 | 38.9 | 42.4 |
| 4.5 | 274 | 27.9 | 40.6 |
| 5.5 | 173 | 7.3 | 24.4 |
| 6.5 | 88 | 1.1 | 6.9 |
| 7.5 | 85 | 1.0 | 6.3 |
| 8.5 | 98 | 1.3 | 6.5 |
| 9.5 | 117 | 4.0 | 20.7 |
| 10.5 | 113 | 5.1 | 25.8 |
| 12 | 127 | 4.3 | 21.9 |
| 14 | 98 | 1.3 | 8.1 |
| 16 | 77 | 1.1 | 7.2 |
| 18 | 64 | 0.9 | 5.8 |
| 20 | 60 | 0.8 | 4.9 |
| 22 | 67 | 1.0 | 4.8 |
| 24 | 48 | 0.6 | 4.7 |

**Table S6.** Saturation index (SI) for vivianite with depth for Group 1 sites. SI was calculated based on measured pore water alkalinity, pH, and concentrations of major ions including Fe^2+^ and P. SI was calculated in PHREEQC using the LLNL database and adjusted vivianite solubility constant at 9°C of log*K*_sp_ = -10.89, which was calculated using the analytical relationship between T and log*K*_sp_ from Al-Borno and Tomson (1994) for Fe_3_(PO_4_)_2_*8H_2_O + 2H^+^ → 3Fe^2+^ + 2HPO_4_^2-^ + 8H_2_O. n.c. = not calculated, Fe^2+^ or P below detection limit.

| **US2 June 2009** | | **US5b Oct 2008** | | **US5b June 2009** | | |
| --- | --- | --- | --- | --- | --- | --- |
| **Depth (cm)** | **SI** | **Depth (cm)** | **SI** | | **Depth (cm)** | **SI** |
| 0 | -5.4 | 0 | n.c. | | 0 | -3.3 |
| 0.25 | -4.6 | 0.5 | -4.9 | | 0.25 | -3.2 |
| 0.75 | -4.5 | 0.75 | -4.5 | | 0.75 | -2.3 |
| 1.25 | -4.8 | 1.25 | -3.9 | | 1.25 | -3.1 |
| 1.75 | -4.7 | 1.75 | -1.2 | | 1.75 | -2.0 |
| 2.5 | -3.9 | 2.5 | 2.9 | | 2.5 | -1.9 |
| 3.5 | -4.8 | 3.5 | 2.5 | | 3.5 | 3.0 |
| 4.5 | -4.5 | 4.5 | 0.9 | | 4.5 | 2.7 |
| 5.5 | -1.0 | 5.5 | -0.1 | | 5.5 | 3.2 |
| 6.5 | 1.7 | 6.5 | -0.6 | | 6.5 | 3.4 |
| 7.5 | 2.3 | 7.5 | -0.3 | | 7.5 | 0.6 |
| 8.5 | 2.4 | 8.5 | 1.8 | | 8.5 | 1.9 |
| 9.5 | 2.5 | 9.5 | 3.9 | | 9.5 | 2.1 |
| 11 | 2.1 | 13 | 4.7 | | 11 | 1.4 |
| 13 | 1.4 | 15 | 5.1 | | 13 | 0.5 |
| 15 | 0.9 | 17 | 4.7 | | 15 | 1.5 |
| 17 | 1.6 | 19 | 4.1 | | 17 | 2.9 |
| 19 | 2.3 | 21 | 4.7 | | 19 | 3.0 |
| 21 | 2.9 | 25 | 4.2 | | 21 | 3.3 |
| 24 | 3.5 | 27 | 3.7 | | 23 | 3.1 |

**Table S7.** Saturation index (SI) for vivianite with depth for Group 2 sites. For calculation details, see caption to Table S6. n.c. = not calculated, Fe^2+^ or P below detection limit.

| **SR5 June 2009** | | **F26 Oct 2008** | | **F26 June 2009** | | |
| --- | --- | --- | --- | --- | --- | --- |
| **Depth (cm)** | **SI** | **Depth (cm)** | **SI** | | **Depth (cm)** | **SI** |
| 0.25 | -7.0 | 0 | -2.5 | | 0 | n.c. |
| 0.75 | -6.3 | 1 | n.c. | | 1 | n.c. |
| 1.25 | -6.3 | 2 | -1.1 | | 2 | n.c. |
| 1.75 | -6.1 | 3 | n.c. | | 3 | n.c. |
| 2.5 | -5.9 | 4 | 2.8 | | 4 | n.c. |
| 3.5 | -5.2 | 5 | 5.9 | | 5 | 0.7 |
| 4.5 | -5.7 | 6 | 6.0 | | 7 | 2.9 |
| 5.5 | -5.3 | 8 | 5.5 | | 9 | 3.0 |
| 6.5 | -0.7 | 10 | 5.8 | | 11 | 2.9 |
| 7.5 | 0.5 | 12 | 5.3 | | 13 | 3.7 |
| 8.5 | 1.2 | 14 | 5.3 | | 15 | 4.1 |
| 9.5 | 1.7 | 17 | 5.4 | | 18 | 4.4 |
| 11 | 1.4 | 19 | 5.4 | | 21 | 4.5 |
| 13 | 2.4 | 30 | 5.6 | | 24 | 4.7 |
| 15 | 2.1 | 35 | 5.1 | | 27 | 4.6 |
| 17 | 1.9 | 38 | n.c. | | 30 | 4.8 |
| 19 | 2.7 |  |  | | 36 | 4.5 |
| 21 | 2.7 |  |  | |  |  |
| 23 | 2.7 |  |  | |  |  |
| 25 | 2.8 |  |  | |  |  |
| 27 | 2.9 |  |  | |  |  |
| 30 | 3.3 |  |  | |  |  |

**Table S8.** Saturation index (SI) for vivianite with depth for Group 3 sites. For calculation details, see caption to Table S6. n.c. = not calculated, Fe^2+^ or P below detection limit.

| **SR1a Oct 2008** | | **SR7 Oct 2008** | |
| --- | --- | --- | --- |
| **Depth (cm)** | **SI** | **Depth (cm)** | **SI** |
| 0 | -7.6 | 0 | -8.9 |
| 1 | -7.2 | 0.5 | n.c. |
| 2 | -7.1 | 1 | n.c. |
| 3 | -5.3 | 4 | -8.3 |
| 4 | -5.9 | 7 | -5.6 |
| 6 | -4.5 | 8 | -4.7 |
| 11 | n.c. | 11 | -5.9 |
| 13 | -3.2 | 14 | -3.1 |
| 18 | -4.2 | 17 | -3.3 |
|  |  | 20 | -2.4 |
|  |  | 26 | -2.3 |
|  |  | 30 | -1.5 |
